# Comparative analysis of 105 datasets across species and tissues reveals differential transcriptomic responses to cannabinoids THC and CBD

**DOI:** 10.1186/s42238-025-00361-0

**Published:** 2025-12-16

**Authors:** Ruoshui Liu, Thomas Kowal, Caden Chow, Tyler Olson, Emily Nguyen, Sen Yang, Jimin Lee, Hua Cai, Xia Yang, Montgomery Blencowe

**Affiliations:** 1 Department of Integrative Biology and Physiology, University of California, Los Angeles, Los Angeles, CA 90095 USA; 2 Interdepartmental Program of Bioinformatics, University of California, Los Angeles, Los Angeles, CA USA; 3 Environmental and Molecular Toxicology Interdepartmental PhD Program, University of California, Los Angeles, Los Angeles, CA USA; 4 Department of Anesthesiology, University of California, Los Angeles, Los Angeles, CA USA; 5 Department of Molecular and Medical Pharmacology, University of California, Los Angeles, Los Angeles, CA USA

**Keywords:** Delta-9-tetrahydrocannabinol, THC, Cannabidiol, CBD, Endocannabinoids system, Transcriptomics, Cannabinoids, Mammalian, Multispecies analysis, Cross-tissue analysis

## Abstract

**Background:**

Cannabis use is on the rise yet the systematic molecular impact of key cannabinoid components on various tissues in diverse organisms remains incompletely understood. We aim to systematically elucidate the molecular pathways and networks affected by delta-9-tetrahydrocannabinol (THC) and cannabidiol (CBD) across species and tissue types.

**Methods:**

We curated 105 THC- and CBD-related RNA sequencing (RNAseq) and microarray datasets from Gene Expression Omnibus (NCBI GEO) with a focus on mammalian species (human, non-human primate rhesus macaque, mouse, rat). Differentially expressed genes (DEGs) were identified using *limma* for microarrays and *DESeq2* for RNAseq data, followed by a meta analysis to identify meta-DEGs. DEGs were analyzed for pathway enrichment using EnrichR, network regulation using Mergeomics key driver analysis, and disease associations using Mergeomics Marker Set Enrichment Analysis. Comparative analyses were conducted across compounds, datasets, species, and tissues.

**Results:**

CBD datasets demonstrated more DEGs and enriched pathways across species and experimental conditions compared to THC. CBD datasets clustered more tightly by route of administration and species and were more frequently enriched for pathways related to zinc homeostasis, inflammation suppression, and cell cycle regulation. In contrast, THC signatures were more heterogeneous and did not exhibit consistent clustering, although consistently altered genes associated with antioxidant activity, neuronal myelination, synaptic signaling, and transcriptional regulation were identified across datasets. THC altered endocannabinoid signaling genes more often in brain tissues, while CBD affected this pathway more heavily in both central and peripheral tissues. Disease enrichment analyses revealed significant associations of CBD DEGs with lipid metabolism and body composition traits, while DEGs of both compounds showed links to neuropsychiatric disorders and type 2 diabetes.

**Conclusions:**

THC and CBD demonstrated distinct and largely non-overlapping transcriptomic responses, with CBD showing more coherent molecular effects across datasets. Our results underscore the potential therapeutic relevance of CBD to metabolic and psychiatric regulation, highlight the context-dependency of THC’s molecular actions, and offer molecular insights into the therapeutic and side effects of cannabinoids.

**Supplementary Information:**

The online version contains supplementary material available at 10.1186/s42238-025-00361-0.

## Introduction

Cannabis use for recreational and medicinal purposes has increased substantially over the past several years in the United States, likely due to broadening legalization of cannabis. By December 2018, medical cannabis was legalized in 33 states and D.C., while recreational use was permitted in 10 states and D.C. (Smart and Pacula 2019). In 2022, 30.7% of US high school seniors reported cannabis use in the past year, with 6.3% using it daily (Miech et al. 2023). Among adults, cannabis use increased from 7.59% to 15.11% in 2013–2022 (Mattingly et al. 2024). Delta-9-tetrahydrocannabinol (THC) and cannabidiol (CBD) are the two most prominent cannabinoids in *Cannabis sativa*, comprising up to 40% of the plant’s extract (Maroon and Bost 2018; Pourseyed Lazarjani et al. 2020).These compounds interact with the endocannabinoid system, a complex network of receptors and signaling molecules that regulate pain, mood, inflammation, and immune responses (Pertwee 2006). THC, the primary psychoactive component, exhibits therapeutic effects as an analgesic for cancer-related chronic pain and has demonstrated anti-invasive and anti-metastatic properties in cancer treatment (Pagano et al. 2022). CBD is non-psychoactive but still influences the brain and nervous system (Batalla et al. 2021; Malabadi et al. 2023), is recognized for its potential in managing epilepsy, anxiety, and neurodevelopmental disorders, and is valued for its anti-inflammatory and antioxidant properties (Machado Bergamaschi et al. 2011).

While cannabinoids have demonstrated therapeutic potential, concerns regarding their safety in terms of physical health, mental health, public safety, and the side effects have tempered their widespread acceptance (Machado Bergamaschi et al. 2011; Sachs et al. 2015). For instance, while some studies suggest that chronic daily use of up to 1500 mg/day is well tolerated in humans, others report both physical and mental side effects such as cognitive impairment, cardiovascular complications, and respiratory issues (Sachs et al. 2015). Additionally, cannabinoids have been linked to immune suppression, resulting in increased susceptibility to human immunodeficiency virus—1 (HIV-1) infections and disease progression. Mental health effects are particularly concerning, as cannabinoids may exacerbate bipolar disorder in predisposed individuals and increase the risk of temporary psychosis. In addition, cannabinoids also pose broader public health and safety concerns. Driving under the influence is associated with impaired motor function and a higher incidence of motor vehicle accidents. Frequent and heavy cannabis use during adolescence increases the risk of developing cannabis use disorder (CUD) (Zuo et al. 2022), while addiction and cannabis dependence may contribute to lower income, unemployment, and reduced life satisfaction (Sachs et al. 2015).

**Fig. 1:**
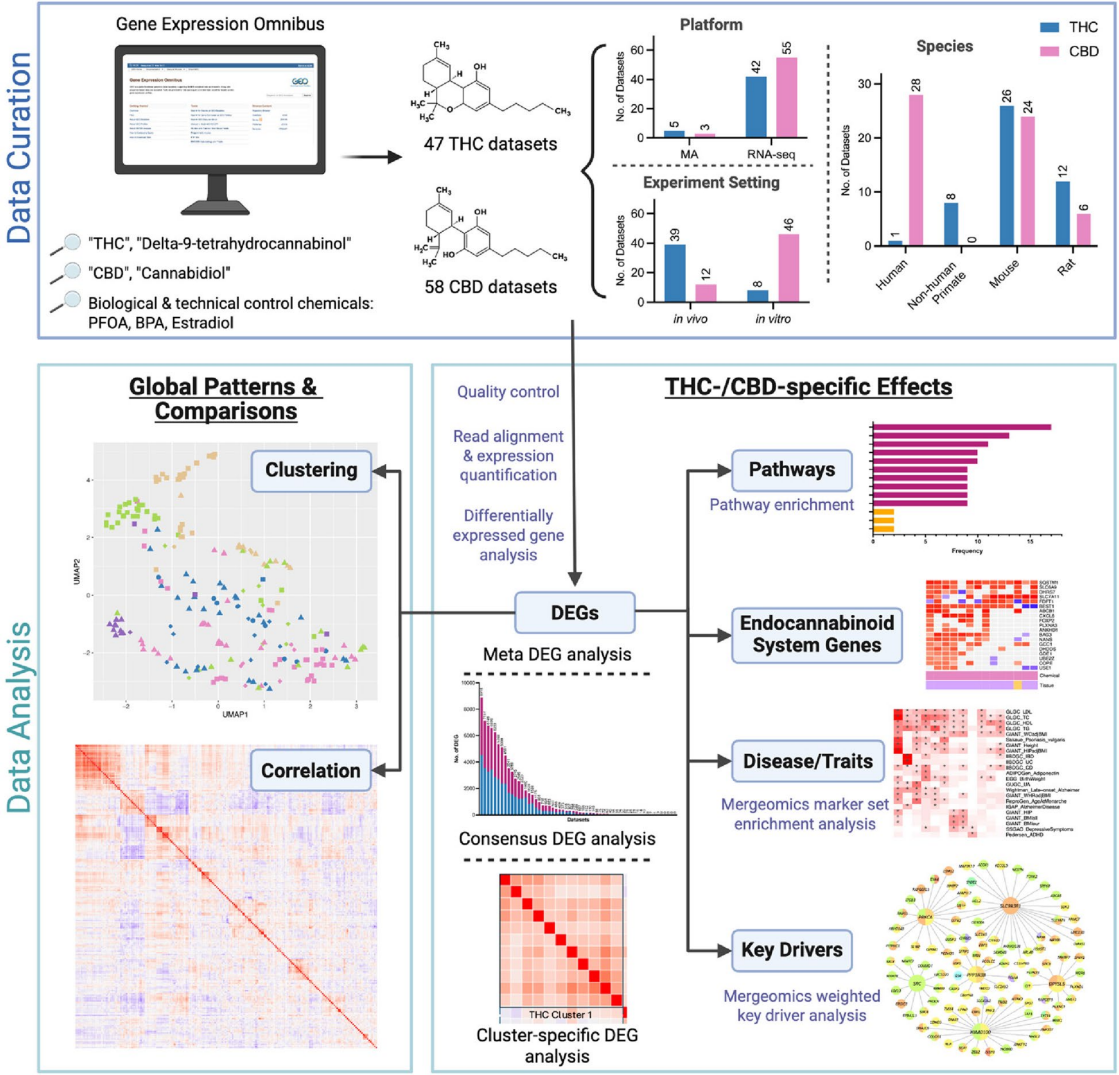
Overall study design. Datasets from THC and CBD exposure studies were curated from GEO and analyzed according to our established pipelines depending on whether they were cDNA microarray or RNAseq studies to detect differentially expressed genes (DEGs) across 4 mammalian species. Control chemicals, including PFOA, BPA, and estradiol, were processed similarly. Gene expression signatures were clustered using dimensionality reduction analysis and correlated using heatmaps derived from Spearman’s rank correlation coefficients. DEGs were then subjected to DEG meta-analysis, biological pathway enrichment, disease enrichment using Mergeomics, and key regulators in brain gene regulatory networks

Therefore, understanding the precise molecular and biological mechanisms underlying the beneficial and adverse effects of THC and CBD is crucial for guiding safe use and developing strategies to mitigate health concerns. At the transcriptomic level, studies have shown that cannabinoid-induced gene expression changes vary depending on tissue type, sex, age, and genetic backgrounds, such as gene mutations (Zuo et al. 2022; Puighermanal et al. 2024; Shapira et al. 2023; Bilkei-Gorzo et al. 2017). This variability has made it challenging to establish precision targets that differentiate the therapeutic vs adverse effects. This study systematically analyzes over 100 transcriptomic datasets related to THC and CBD across species and tissue types from the Gene Expression Omnibus (GEO) (Edgar et al. 2002) (Fig. 1). Using transcriptomic data, we identified differentially expressed genes (DEGs) for each dataset and derived meta-DEGs for each cannabinoid through meta-analysis. We further investigated global regulatory patterns across datasets through clustering and correlation assessment as well as cannabinoid-specific effects through pathway and disease association analysis and network modeling. We also investigated the effects of THC and CBD on the endocannabinoid system across the datasets. These integrative approaches allowed us to uncover consistent and unique patterns of gene regulation across studies to partition potential targets underlying beneficial vs. adverse effects of THC and CBD.

## Materials & methods

### Data curation

Transcriptomic data from previous studies were curated from the National Center for Biotechnology Information’s Gene Expression Omnibus (NCBI GEO) (Edgar et al. 2002; Barrett et al. 2012). To identify THC-specific datasets, we queried GEO using the keywords “THC” and “Delta-9-tetrahydrocannabinol,” while CBD-specific datasets were retrieved using “CBD” and “Cannabidiol.” Studies were included if they met the following criteria: 1) the model organism was human, mouse, rat, or rhesus macaque to focus on mammalian species with higher translational potential; 2) the dataset was generated using cDNA microarrays or RNA sequencing; 3) a minimum sample size threshold of *n* = 3 per group for in vivo studies and *n* = 2 per group for in vitro studies was applied, allowing us to include most available studies while balancing data quality and comprehensiveness; 4) the study was not part of a subseries or superseries on GEO to avoid duplicate data. For studies testing multiple conditions, samples were grouped based on their matching physiological and pathological background conditions to ensure accurate differential expression analysis. Specifically, only control and treatment samples with shared conditions, such as prior chemical exposures, genetic mutations, and preexisting diseases, were analyzed together. This approach allowed for the assessment of treatment effects within comparable biological contexts while minimizing confounding factors. A total of 47 datasets for THC and 58 datasets for CBD met the criteria and were curated (Table S1).

To validate that our data curation and analysis procedures can identify THC and CBD-specific transcriptomic effects, we also included additional well-studied, non-cannabinoid chemicals, including bisphenol A (BPA), perfluorooctanoic acid (PFOA), and estradiol, for comparison. Our previous studies of BPA and PFOA have revealed stable and consistent gene signatures for PFOA but more variable gene expression changes in response to BPA across studies, making them potential reference points to assess gene signature stability vs variability (Zamora et al. 2024; Diamante et al. 2021; Chen et al. 2016). We also included estradiol, an endogenous bioactive molecule with well-known biology, as a control to assess whether our analytical pipeline retrieves known biology. Gene expression data for these chemicals were obtained from GEO and processed using the same methods as described for THC and CBD. A total of 50 BPA datasets, 39 estradiol datasets, and 14 PFOA datasets were curated (Table S2).

### Data download and preprocessing

Microarray data were downloaded from GEO using the *GEOquery* package (Davis and Meltzer 2007). As microarray data submitted to GEO are pre-processed and quality-controlled, we verified normalization and applied log2 transformation before downstream analysis.

Raw RNA sequencing (RNA-seq) datasets were retrieved from NCBI’s Sequence Read Archive (SRA), quality-checked, and processed (Leinonen et al. 2010). FASTQ files were downloaded using the parallel-fastq-dump wrapper, followed by quality control and preprocessing. Trim Galore (v0.6.6) was used to remove low-quality bases from the 3’ end of reads (2015). Cutadapt (v2.1.0) (Martin 2011) was employed to remove adapter sequences. Reads shorter than 20 base pairs after trimming and adapter removal were filtered out to ensure data quality.

Reads were mapped to appropriate species-specific reference genomes using Salmon (v 1.9.0) (Patro et al. 2017). The reference genome assemblies used were GRCh38.108 for *Homo sapiens*, GRCm39.108 for *Mus musculus*, mRat.BN7.2.108 for *Rattus norvegicus*, and Mmul_10.108 for *Macaca mulatta*. Reference genomes for each species were indexed using the Salmon index tool to optimize alignment efficiency. Finally, transcript-level quantification results from Salmon were imported and summarized using the *tximport* package (v1.32.0) (Soneson et al. 2016).

### Differentially expressed gene (DEG) analysis

For each dataset, the treatment group (THC or CBD) was compared to its corresponding control group to identify DEGs using Linear Models for Microarray Data (*LIMMA*) for cDNA microarray datasets (Ritchie et al. 2015) and *DESeq2* for RNA-seq datasets (Love et al. 2014). Multiple testing was corrected using the Benjamini-Hochberg (BH) method to obtain false discovery rate (FDR). For studies with multiple doses, baseline conditions (e.g. prior chemical exposures, genetic mutations, and preexisting diseases), or time points, each variation was treated as an independent dataset to extract distinct DEG signatures for each dose, condition, and timepoint. DEGs were considered significant at an FDR < 5%. All gene labels were converted to their human orthologs to facilitate cross-species comparisons.

### Clustering and correlation analysis of DEG gene signatures across datasets

To compare DEGs across studies, we combined the gene expression log fold changes from individual studies from the differential gene expression analysis. Since RNAseq and microarray experiments can produce log fold changes on different scales, we applied a rank-based normalization method to standardize values within a range of −1 (downregulated in the treatment group) to 1 (upregulated in the treatment group) for each dataset. Specifically, we separately ranked positive and negative log fold changes within each dataset. Positive values were ranked in ascending order and scaled between 0 and 1 (no change to most upregulated), while negative values were ranked in descending order and scaled between −1 and 0 (most downregulated to no change). This approach preserves the relative magnitude of gene expression changes among genes while making the datasets comparable across studies and transcriptome platforms. To assess similarity and differences across datasets for THC and CBD through cluster analysis, we used the top 2,500 most variable genes. For the analysis across all chemicals (THC, CBD, PFOA, BPA, estradiol), given the large number of datasets, we first selected genes present in at least 70% of the 208 datasets before selecting the top 2,500 most variable genes. Missing values in the DEG fold change table were imputed using the *missForest* R package (Stekhoven and Bühlmann 2012).

#### UMAP non-linear dimensionality reduction to visualize dataset similarity

We applied UMAP for non-linear dimensionality reduction using Euclidean distance, which measures proximity or similarity between datasets and identify nearest neighbors to visualize the similarity patterns across chemicals and datasets. It has been widely used in single-cell RNA-seq analyses to visualize cells with similar gene expression patterns to define cell types and in bulk transcriptome meta-analysis to identify data clusters with consistent underlying biological features (Yang et al. 2021). In our study, UMAP serves as a complementary approach to visualize data similarity patterns and corroborate dataset clusters identified through correlation-based clustering below.

#### Spearman correlation and hierarchical clustering

As an alternative approach to assess similarity and differences across datasets, we used Spearman’s rank correlation analysis of the normalized gene expression fold change data to compute pairwise correlations among datasets. Dataset clusters were subsequently defined through hierarchical clustering based on the correlation dendrograms (i.e., datasets were defined to be in a cluster only if they shared the same dendrogram branch below a height cutoff greater than 2) and a requirement for a dominance of positive correlations between datasets within each cluster.

### Consistent DEG analysis

To identify genes consistently differentially expressed across datasets within each cluster and across all datasets in response to THC or CBD, we performed a meta-analysis using the Robust Rank Aggregation (RRA) package (v1.2.1) (Kolde et al. 2012). For cluster-level analyses, rank aggregation was conducted separately for up- and downregulated genes within each dataset cluster, whereas for the overall analysis, rank aggregation was applied across all DEGs from all datasets. Genes with an RRA score < 0.05 were considered robust, consistent DEGs, reflecting consistent differential expression across datasets within each cluster. The RRA score measures the probability of a gene achieving its observed ranking pattern across datasets by chance, with lower scores indicating greater consistency.

### Pathway enrichment analysis

Pathway enrichment analysis was performed on the identified DEGs using EnrichR (Chen et al. 2013). DEGs were compared against the Gene Ontology Biological Process (GOBP) (Ashburner et al. 2000) databases to identify significantly enriched pathways. Pathways with an FDR < 5% and at least 5 overlapping DEGs were considered statistically significant.

### Weighted Key Driver Analysis (wKDA) for brain network modeling of THC and CBD DEGs

To identify potential gene regulatory networks and network key drivers (KDs) underlying cannabis-induced brain effects, we applied Weighted Key Driver Analysis from the Mergeomics pipeline (Ding et al. 2021; Shu et al. 2016). We conducted the analysis separately from THC- and CBD-treated brain datasets. Using a previously constructed brain Bayesian network based on large human and animal model omics studies in Mergeomics, we identified nodes (genes) whose immediate subnetworks were enriched for the THC or CBD DEG sets at FDR < 0.05 as statistically significant KDs. Network visualizations were performed using Cytoscape (Shannon et al. 2003).

### Marker Set Enrichment Analysis (MSEA) for disease/trait association assessment

MSEA in the Mergeomics package was used to identify the enrichment of THC or CBD DEGs for genetic associations with 101 genome-wide association studies (GWAS) of diseases and phenotypic traits (Ding et al. 2021; Shu et al. 2016). Disease-associated genes were mapped using full summary statistics from the GWAS Catalog (MacArthur et al. 2017), with single nucleotide polymorphisms (SNPs) assigned to genes within a 50 kb distance. MSEA employs a chi-square-like statistic with multiple quantile thresholds to assess whether a DEG set shows enrichment of disease SNPs compared to random chance. 10,000 permuted gene sets were generated for each DEG set. As detailed in Shu et al. (Shu et al. 2016), the enrichment statistics from the permutations were used to approximate a Gaussian distribution from which enrichment *p*-values were determined. FDR was estimated using the BH correction. DEG sets were determined to be statistically significant for a given disease or trait if FDR < 5%.

## Results

### Curation of transcriptomic datasets on cannabinoids across species and tissues

To investigate the impact of cannabinoids on gene expression, we obtained 108 transcriptomic datasets from GEO (Edgar et al. 2002) (Table S1). Specifically, we obtained 3 datasets for overall cannabis use, 58 for CBD, and 47 for THC, spanning 32 human (*Homo sapiens*) studies, 8 non-human primate (*Macaca mulatta*) studies, 50 mouse (*Mus musculus*) studies, and 18 rat (*Rattus norvegicus*) studies across 15 broad tissue categories (e.g. blood, brain, cancer, digestive, heart, immune, kidney, liver, lung, muscle, oral, placenta, skin, stem cell, and vasculature). Since the “overall cannabis use” datasets involved either cannabis smoke exposure or observational studies in which other botanical compounds from the cannabis plant were not controlled for, we excluded them from our analysis to maintain our specific focus on the compounds THC and CBD. Notably, reflecting the focus on neurological effects of cannabinoids, brain-related datasets accounted for nearly half of all studies.

Many studies included various physiological and pathological background conditions in both control and treatment groups, such as genetic mutations (e.g., *Cox15*, *Ndufs4*), preexisting diseases (e.g., SARS-CoV-2, Simian Immunodeficiency Virus (SIV) infection), and prior chemical exposures (e.g., 2'−3'-cGAMP, formoterol/budesonide treatment). Additionally, the datasets covered variations in study design, including differences in sex distribution, dose, route and duration of administration. The broad coverage of diverse conditions allows us to not only determine specific transcriptomic signatures for each condition but also assess the consistency of cannabinoid-specific effects across datasets and evaluate study design factors that influence transcriptomic effects. After downloading and preprocessing the gene expression data, we conducted differential gene expression, pathway enrichment and disease association analyses to characterize the transcriptomic effects of cannabinoids (Fig. 1).

### THC and CBD transcriptomic effects are distinct from other chemicals

To evaluate whether cannabinoids THC and CBD show distinct transcriptomic signatures compared to other chemicals, we incorporated 103 datasets from three well-characterized chemicals, Bisphenol A (BPA), perfluorooctanoic acid (PFOA), and estradiol, which have been associated with metabolic and reproductive functions, respectively. Both BPA and PFOA are endocrine-disrupting chemicals that influence cardiometabolic disease-related pathways (Zamora et al. 2024; Diamante et al. 2021; Chen et al. 2016). Estradiol, a primary estrogen hormone, plays a role in reproductive and sexual function by regulating gene expression through estrogen receptors (Kovács et al. 2020; McCarthy 2008). We selected these as comparative datasets because we expect them to be functionally distinct from THC and CBD. In total, we included 50 BPA datasets, 39 estradiol datasets, and 14 PFOA datasets from GEO (Table S2) and processed them using the same methodology as for the cannabinoids.

**Fig. 2:**
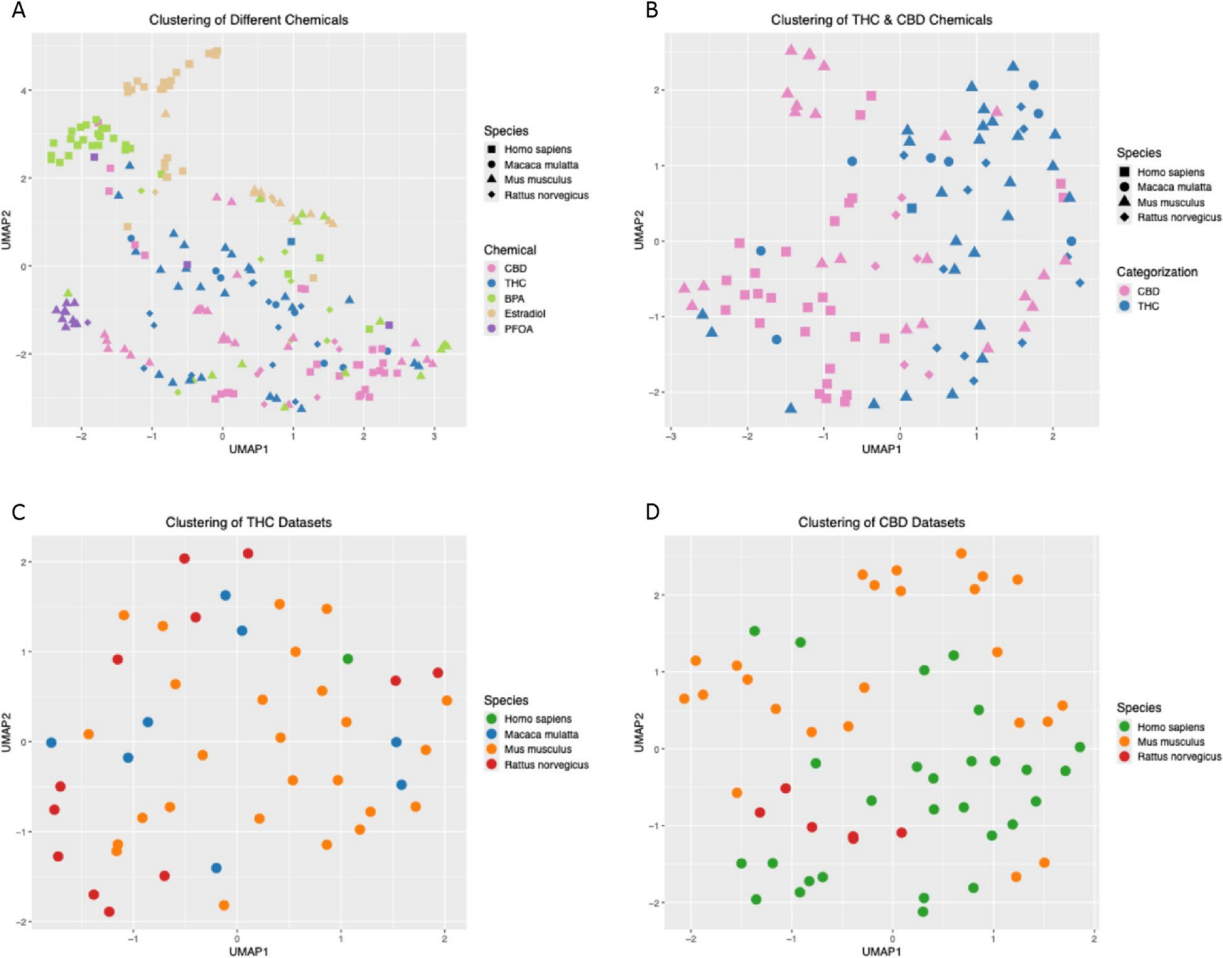
UMAP clustering reveals instability in DEG signatures across tissues in THC and CBD. Gene expression signatures were clustered using UMAP by the normalized log2 fold change induced in each gene between the treatment and control groups for THC and CBD, as well as the reference endocrine disrupting chemicals PFOA, BPA, and estradiol. Across all these chemicals, the top 2,500 most variable genes by log2 fold change were included in this analysis. Dots in the plot correspond to individual DEG signatures. In **A**-**B** the color of the dot corresponds to the chemical, and the shape of the dot corresponds to the species. **A** PFOA (purple), BPA (green), and estradiol (yellow) all form relatively tight clusters on the left-hand side of the plot. THC (blue) and CBD (pink) signatures are more widely spread, indicating a higher degree of variability in the DEG signatures curated for this study. **B** THC (blue) and CBD (pink) exhibit subtle separation along the UMAP1 dimension, indicating weak differences between these two chemicals in their DEG signatures. In **C**-**D** the color of the dot corresponds to the species. **C** Within THC signatures, signatures derived from experiments on all four model organisms clustered together with no sharp distinction among any of them, revealing that none of the species has a species-specific response in their THC-associated DEG signatures. **D** Within CBD signatures, mouse datasets and human datasets show subtle shifts along the UMAP2 dimension, and rat datasets cluster tightly, revealing that CBD exhibits weak species-specific effects on gene signatures

We first compared the chemical-induced expression changes for all genes across all THC, CBD, BPA, estradiol, and PFOA datasets by performing dimensionality reduction analysis using UMAP on the normalized log2 fold changes of all genes. Agreeing with our hypothesis, most BPA, estradiol, and PFOA datasets clustered closer to one another within each chemical but further away from datasets of other chemicals in UMAP, supporting biological similarity of datasets within each chemical and differences between chemicals (Fig. 2A). These results suggest that comparative transcriptomics analysis captures gene expression patterns that likely reflect the different molecular mechanisms of different classes of chemicals. Agreeing with our previous observation on higher consistency of PFOA gene signatures (Zamora et al. 2024), most PFOA datasets clustered tightly in UMAP. In comparison, the distance between datasets for BPA or estradiol was larger than that for PFOA. Compared to these reference chemicals, THC and CBD datasets were more spread out in UMAP, suggesting less coherent transcriptomic changes across THC and CBD datasets. Additionally, datasets from RNA sequencing and microarray platforms did not show separation in UMAP, indicating a minimal effect of the technical platform on gene signatures (Fig. S1A).

We also observed that some datasets from the same study or experimental setup clustered closer, such as the BPA and estradiol microarray datasets from GSE50705, which were clustered in the upper left corner of the UMAP plot (Fig. 2A). To mitigate the contribution of study-specific batch effects to the UMAP patterns, we used the average log fold changes of each gene across datasets for each chemical from the same study for dimensionality reduction again (Fig. S1B). The UMAP patterns remained consistent, confirming that study-related effects did not drive the observed trends in Fig. 2A: PFOA exhibited the highest similarity across datasets, followed by estradiol and BPA, while CBD and THC datasets were more dispersed in UMAP.

### CBD gene signatures demonstrate tighter clustering across species and routes of administration compared to THC

To better assess the similarities and differences between THC and CBD datasets, we further clustered THC and CBD datasets excluding BPA, estradiol, and PFOA datasets. There was a subtle separation between THC and CBD datasets along the first UMAP dimension (UMAP1), indicating that their global transcriptional effects exhibit certain differences, however they were not strongly distinguishable (Fig. 2B). Notably, datasets from both RNAseq and microarray platforms were mixed, again supporting that the technological platform was not a major contributor to the UMAP patterns (Fig. S1C, S1D, S1E).

Within the THC datasets, we found no distinct clustering patterns based on species (Fig. 2C), route of administration (Fig. S1F), or tissue type (Fig. S1G), suggesting that these factors did not significantly influence global transcriptional profiles.

In contrast, CBD datasets exhibited weak species-specific clustering. Along the second UMAP dimension (UMAP2), *Homo sapiens* and *Mus musculus* datasets showed subtle shifts, whereas *Rattus norvegicus* datasets clustered more closely (Fig. 2D). Regarding the route of administration, in vitro (in medium) datasets tended to cluster at higher UMAP1 values, whereas in vivo (injection or ingestion) datasets were mainly positioned at lower UMAP1 values (Fig. S1H). In the tissue-based UMAP (Fig. S1I), no clear clustering trends emerged across tissue types. However, datasets from the same study, such as the two heart datasets and two kidney datasets, clustered more closely, suggesting that study-specific effects contributed to their proximity.

Overall, the weak clustering patterns across species, tissues, and routes of administration in UMAP suggest that these factors did not play a dominant role in shaping the global transcriptomic shifts induced by THC or CBD, but certain weak patterns were observed for CBD datasets.

### CBD studies demonstrate different correlation patterns than THC datasets

In addition to analyzing dataset similarity patterns through dimensionality reduction using UMAP, we assessed correlation patterns in gene expression changes across all THC, CBD, BPA, estradiol, and PFOA datasets. A positive correlation (red) indicates that most genes are upregulated or downregulated concordantly between two datasets, whereas a negative correlation (blue) suggests discordance or opposite directions in gene regulation patterns. Results from this analysis are consistent with our UMAP clustering results in that the molecular patterns of THC and CBD are less coherent compared to the reference chemicals (Fig. S2).

**Fig. 3:**
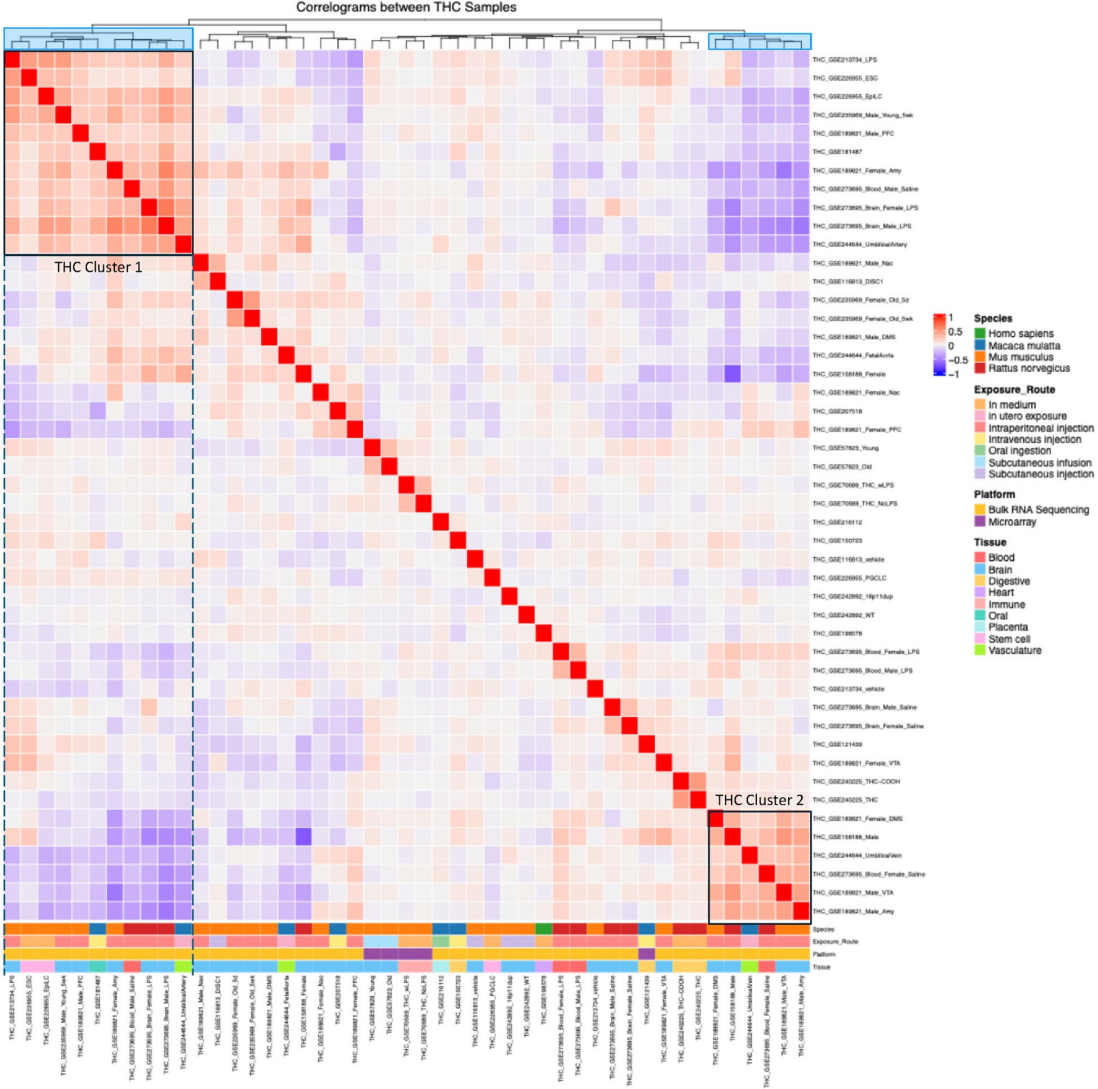
Hierarchical clustering reveals clusters of datasets with concordant gene expression signatures in response to THC exposure. The log2 fold changes across the genome for each THC dataset were used to calculate Spearman’s correlation coefficients between datasets, perform hierarchical clustering analysis, and identify coherent clusters. The results are shown in the heatmap. Red hues indicate concordant gene signatures (e.g. both datasets have their genes changing in the same direction and by a similar magnitude) while blue hues indicate discordant gene signatures. We focus on datasets that are under the same main cluster branch (highlighted in blue in the top cluster dendrogram) and show dominant positive correlations. Two distinct clusters of concordant THC datasets are indicated, with cluster 1 containing 11 datasets in the upper left quadrant and cluster 2 containing 6 datasets in the lower right quadrant. Both clusters are composed mostly of brain-related datasets and contain datasets derived from multiple species. These two clusters exhibited moderate negative correlations with each other

**Fig. 4:**
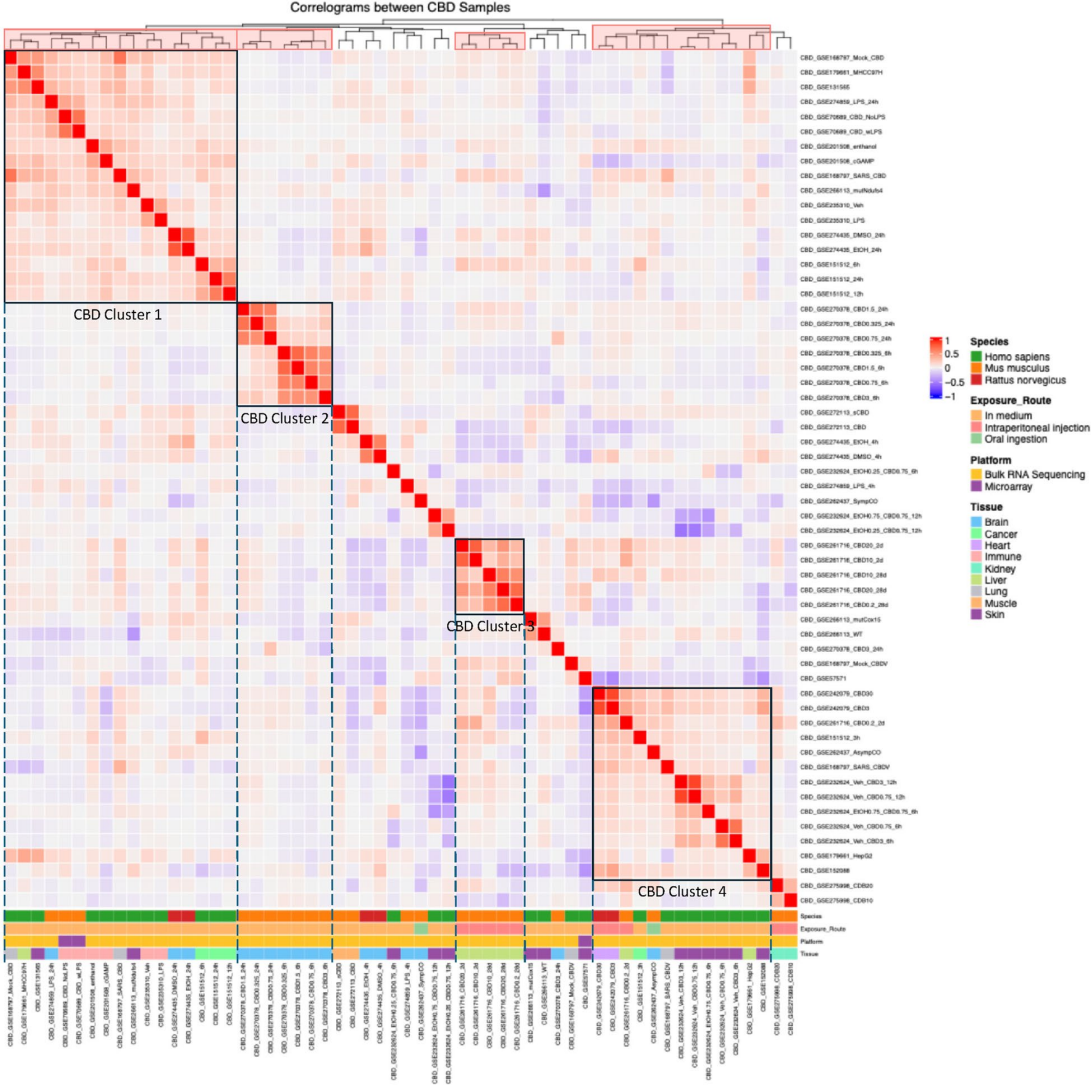
Hierarchical clustering reveals clusters of datasets with concordant gene expression signatures in response to CBD exposure. The log2 fold changes across the genome for each CBD dataset across were used to calculate Spearman’s correlation coefficients and perform hierarchical clustering analysis. The results are shown in the heatmap. Red hues indicate concordant gene signatures (e.g. both datasets have their genes changing in the same direction and by a similar magnitude) while blue hues indicate discordant gene signatures. We focus on datasets that are under the same main cluster branch (highlighted in pink in the top cluster dendrogram) and show dominant positive correlations. Four distinct clusters of concordant CBD datasets are indicated, with cluster 1 containing 17 datasets in the upper left quadrant, cluster 2 containing 7 datasets in the center-left region, cluster 3 containing 5 datasets in the central right region, and cluster 4 containing 15 datasets in the lower right quadrant. Both cluster 1 and cluster 4 contained datasets from multiple species and tissue types. Cluster 1 is purely in vitro datasets where cluster 4 has a mixture of multiple exposure routes and a high representation of skin-derived datasets. Clusters 2 and 3 were composed of datasets derived from one murine hypothalamus study and one murine liver study, respectively

Next, we carried out hierarchical clustering analyses separately for THC and CBD datasets to derive hierarchical correlation dendrograms (Figs. 3 and 4) based on the correlation values in gene expression changes between datasets, which defined multiple dataset clusters. In the THC correlation heatmaps, two major clusters of positively correlated datasets were observed: THC Cluster 1 in the upper left quadrant (11 datasets) and THC Cluster 2 in the lower right quadrant (6 datasets), both largely composed of brain-related datasets spanning multiple species. However, these two clusters exhibited moderate negative correlations with each other (Fig. 3). This result suggests that THC gene signatures from the brain could be partitioned into distinct patterns with each pattern showing consistency across studies. Notably, some datasets with the same experimental designs but differing in sex were assigned to different clusters. For instance, in GSE273695, which analyzed rat blood samples with daily intraperitoneal injections escalating from 2.5 mg/kg to 10 mg/kg, the male dataset clustered in THC Cluster 1, while the female dataset appeared in THC Cluster 2. Additionally, multiple mouse brain datasets from GSE189821 (daily 10 mg/kg intraperitoneal injection) were distributed across both clusters: the male amygdala dataset was placed in THC Cluster 2, while the female amygdala dataset fell into THC Cluster 1. However, this separation of male and female samples into different clusters was not observed in all datasets. For example, rat prefrontal cortex datasets of both sexes in GSE273695 were all assigned to THC Cluster 1. While this finding suggests that sex may influence THC-induced transcriptomic responses, this sex-based separation was not consistently observed across all THC datasets and hence any potential sex-specific effects are likely more nuanced and depending on tissue type or other conditions to be elucidated.

For CBD datasets, hierarchical correlation dendrograms revealed four distinct clusters, with the two largest being CBD Cluster 1 in the upper left quadrant (17 datasets) and CBD Cluster 4 in the lower right quadrant (15 datasets) (Fig. 4). These clusters were primarily composed of in vitro experiments and included datasets from three species: human, mouse, and rat. CBD Cluster 1 featured a mixture of tissues, with prominent representation from immune, cancer, and brain. In contrast, approximately half of the datasets in CBD Cluster 4 were from the skin. Two smaller clusters were observed in the central region of the plot, representing study-and tissue-specific clusters: CBD Cluster 2 included 7 mouse hypothalamus cell line datasets from GSE270378, and CBD Cluster 3 contained 5 mouse liver datasets from GSE261716, where datasets varied by CBD dosage and exposure time.

Overall, the correlation-based clustering analysis of THC and CBD datasets revealed two brain-related dataset clusters for THC and four clusters for CBD that showed distinct patterns of transcriptomic changes.

### CBD induces more significant DEGs than THC

**Fig. 5:**
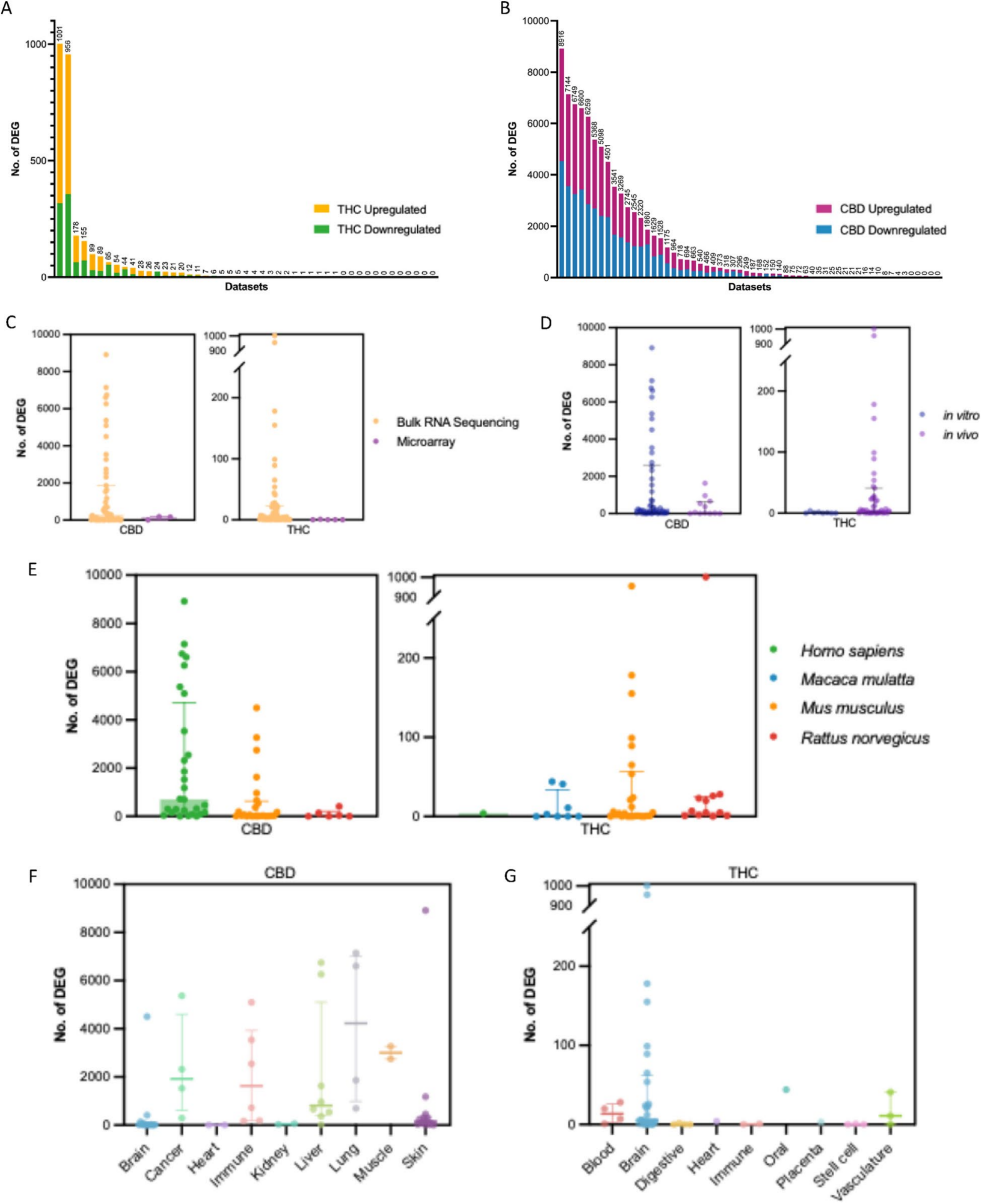
DEG results indicate CBD induces more DEGs across tissues and identify factors affecting the number of observed DEGs across studies. The number of DEGs observed across datasets was quantified according to chemical exposure and the potential confounding variables of sequencing platform, route of exposure, species, and tissue. **A** A bar plot showing the number of DEGs detected in each THC dataset analyzed. Four datasets had hundreds of DEGs and approximately half of the datasets had at least ten, while very low or zero DEG counts were observed in approximately half of the studies. **B** A bar plot showing the number of DEGs detected in each CBD dataset analyzed. Nearly one third of datasets demonstrated more than 1,000 DEGs, and a significant portion of datasets also resulted in hundreds of DEGs. A small number of datasets with ten or fewer DEGs was also observed. **C** A dot plot showing the amount of DEGs observed in each study for CBD (left) and THC (right), colored according to the sequencing platform used to derive each dataset. In general, RNAseq datasets are more sensitive to DEGs, and more are observed in these studies than in cDNA microarray studies of the same chemical exposures. **D** A dot plot showing the amount of DEGs observed in each study for CBD (left) and THC (right), colored according to whether the study design used an in vivo or in vitro exposure model. CBD datasets with in vitro exposure paradigms exhibit more DEGs while THC datasets with in vivo exposures exhibit more DEGs. **E** A dot plot showing the amount of DEGs observed in each study of CBD (left) and THC (right), colored according to the species used in each study. CBD human and mouse datasets exhibited higher numbers of DEGs than rat datasets, while THC mouse datasets exhibited the most DEGs with slightly fewer in rat and macaque models. **F**-**G** Dot plots showing the number of DEGs for CBD (**F**) and THC (**G**) datasets, colored according to the tissue examined in each dataset. CBD exhibited high numbers of DEGs in all tissues except the heart and kidney, with consistently high numbers of DEGs observed in the liver, lung, immune cells, and cancer cell line models across tissues. THC consistently exhibited high numbers of DEGs in brain datasets with far fewer DEGs in the rest of the tissues, suggesting brain-specific gene perturbation effects

To go beyond global transcriptomic patterns and focus on individual genes exhibiting significant changes in response to THC and CBD, we focused to significant DEGs at FDR < 5%. Overall, CBD induced more significant DEGs (thousands; Fig. 5A) than THC (mostly < 100 DEGs; Fig. 5B) (Table S3). Kolmogorov–Smirnov (KS) tests of the distributions of DEG counts across datasets confirmed that both upregulated and downregulated gene counts differed significantly between THC and CBD.

To assess the contribution of the various study design factors to the DEG findings, we assessed the effects of chemical type (THC vs CBD), transcriptome platform (RNAseq vs microarray), species, tissue, exposure type (in vivo vs in vitro), duration, dosage, and sex. Multiway ANOVA analysis showed the strongest significant effect of chemical type (more DEGs for CBD than THC), followed by statistically significant effects of tissue, species, and platform (Table S4). RNA-seq datasets generally yield more DEGs than microarray datasets (Fig. 5C). Both in vitro and in vitro exposure studies of CBD exhibited higher numbers of DEGs than THC (Fig. 5D). Examining species effects in CBD datasets, human in vitro cell line studies had the highest number of DEGs (Fig. 5E). For THC datasets, where the most DEG-rich studies are in vivo, mouse studies generate the largest number of significant DEGs, followed by non-human primate and rat studies. At the tissue level, CBD studies show the highest median number of DEGs in lung, muscle, cancer, immune, and kidney tissues (Fig. 5F). For THC, while no tissue exhibits a notably high median DEG count, the brain had the greatest variation in DEG numbers across studies, likely due to brain region-specific effects (Fig. 5G). Additionally, as expected, the number of DEGs in CBD studies increases with both exposure time and dosage (Fig. S3A, S3B, S3C for different studies). For THC, no study specifically examined both time points and dosage variations. Unlike time and dosage, sex had a notable but inconsistent effect on DEG counts. Among 10 THC datasets with identical experimental designs differing only by sex, females exhibited more DEGs in five datasets, including the mouse brain amygdala, dorsolateral striatum, ventral tegmental area (VTA), prefrontal cortex, and rat peripheral blood mononuclear cells (PBMCs) under LPS stimulation. In contrast, males showed higher DEG counts in four datasets: the rat brain orbitofrontal cortex (under both LPS and saline treatments), rat PBMCs with saline, and the mouse brain nucleus accumbens (Fig. S3D). This variability highlights the complexity of sex-specific factors in cannabinoid responses. Additional sensitivity analysis using subsets of THC and CBD datasets matched by exposure route (Fig. S4A), platform (Fig. S4B), and sex (Fig. S4C) confirmed that the higher number of DEGs for CBD than THC were not due to the study design imbalances between the two chemicals.

To further assess whether the differences in DEG numbers between THC and CBD were a function of sample size and hence statistical power of the curated datasets, we examined the correlation between the sample sizes of each study and the number of DEGs detected. We found no significant correlation between the sample size and DEG number for THC (*r* = 0.035, *p* = 0.817; Fig. S5A), CBD (*r* = −0.021, *p* = 0.873; Fig. S5B), or all datasets combined (*r* = −0.082, *p* = 0.873; Fig. S5C). Therefore, the observed variation in DEG number is not driven by sample size differences within our curated data.

### CBD demonstrates higher consistency at gene and pathway levels across datasets

To evaluate the consistency of DEGs across datasets for each cannabinoid, we applied three complementary approaches: 1. identifying consistent DEGs within correlation-based clusters, 2. assessing individual DEG recurrence and directionality consistency across datasets, and 3. performing meta-analytical aggregation of DEGs across all datasets.

#### Cluster-level consistency

We used rank aggregation to identify consistently upregulated and downregulated DEGs across datasets within each dataset cluster based on hierarchical cluster analysis (Figs. 3 and 4) (Kolde et al. 2012). THC Cluster 1 contained relatively few DEGs, with 29 consistently upregulated genes, 18 consistently downregulated genes, and no significantly enriched pathways (Fig. 3, Table S5). Many consistent DEGs are related to neurodevelopment and brain functions. For example, upregulated genes include *TBR1*, a key regulator of cortical development (Hevner et al. 2001); *NPTX2*, neuronal pentraxin for synaptic plasticity (Zhou et al. 2023); neuropeptide S receptor *NPSR1* in neuroendocrine cells (Pulkkinen et al. 2014); *ADRA2B*, which regulates neurotransmitter norepinephrine (Xie et al. 2018); *ADORA2A*, linked to anxiety, arousal, and sleep regulation (Hohoff et al. 2020). Downregulated genes include *FEZF1* for neuronal differentiation (Shimizu et al. 2010); *LHX5* associated with mammillary body development (Heide et al. 2015); *GPR50* for neural progenitor cell differentiation (zahid Khan and He 2016); *CACNA1B*, a calcium channel subunit for neurotransmitter release (Szymanowicz et al. 2024). THC Cluster 2 showed only two consistently downregulated genes, B Cell receptor *CD72* (Wu and Bondada 2002) and presynaptic receptor *GRM8* (Scherer et al. 1997), and no significant pathways (Fig. 3, Table S6).

In contrast, CBD Cluster 1 exhibited robust transcriptional changes, with 1,005 consistently upregulated DEGs and 997 consistently downregulated DEGs (Fig. 4, Table S7). The upregulated pathways included negative regulation of growth, cellular response to zinc ions, response to ER stress, and inflammatory response. The downregulated pathways were primarily related to development, ECM organization, and sprouting angiogenesis, and skin development. CBD Cluster 4 had 251 consistently upregulated DEGs and 283 downregulated DEGs (Fig. 4, Table S8), but only the upregulated DEGs showed significantly enriched pathways, including negative regulation of growth and cellular response to zinc ions. The absence of strong downregulated pathways in Cluster 4 may explain the separation between the two large clusters and their weak negative correlation. CBD Clusters 2 and 3 displayed more limited consistency. CBD Cluster 2 only had 2 consistent DEGs: upregulation of cellular zinc sensor *MTF1* (Andrews 2001) and downregulation of *SENP3* in *Wnt* signaling (Wang et al. 2024) (Fig. 4, Table S9). CBD Cluster 3 had 73 consistently upregulated DEGs, 77 downregulated DEGs, and two enriched upregulated pathways: cellular response to lipopolysaccharide and inflammatory response (Fig. 4, Table S10).

#### Consistency in DEGs across datasets

**Fig. 6:**
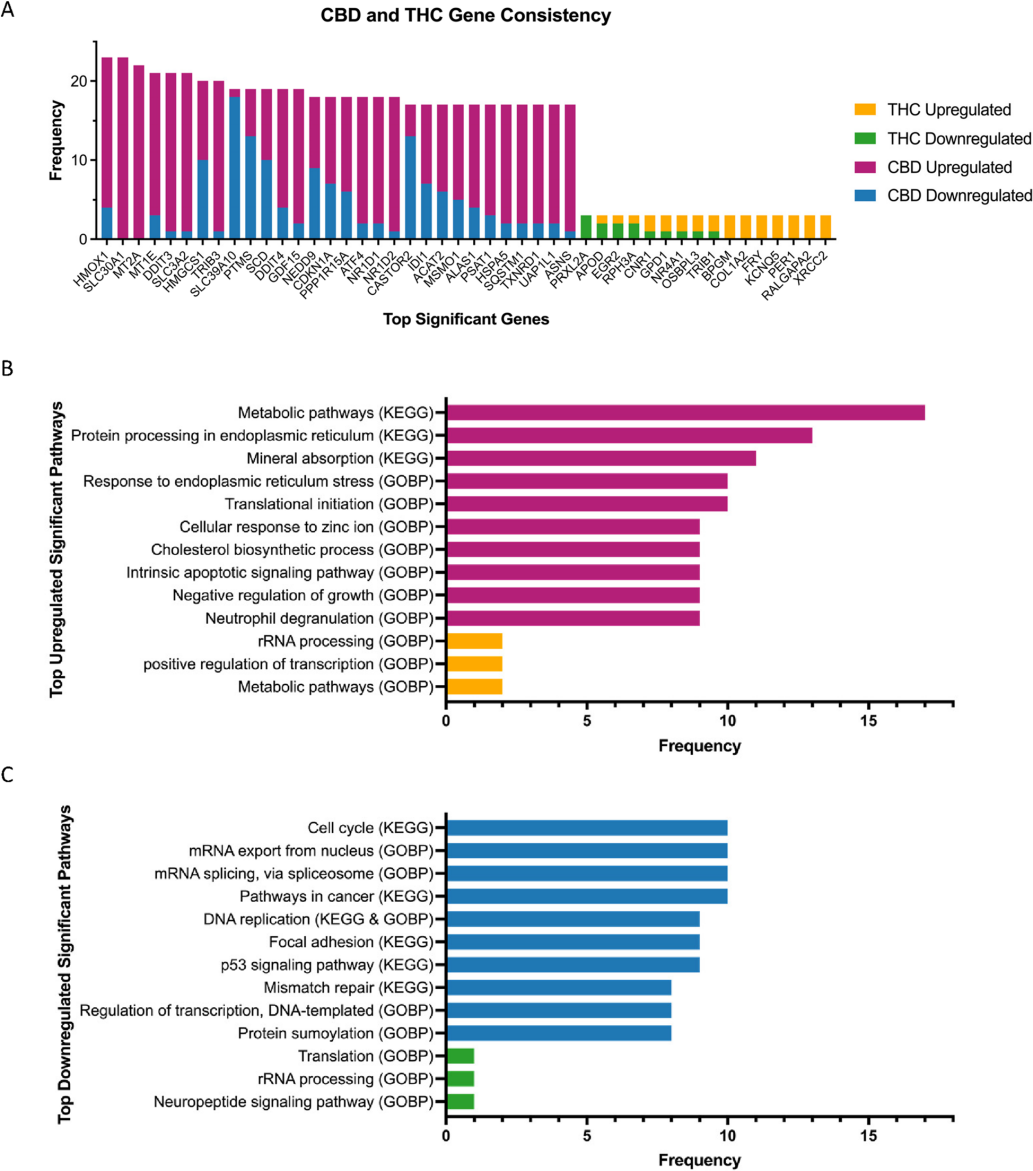
Meta-analysis reveals highly represented gene and pathway perturbations across cannabinoid exposure signatures. **A** Recurrent genes that were detected as DEGs across CBD and THC exposure datasets are plotted on this bar graph. CBD tended to induce more DEGs and thus has recurrent genes that appear more often across datasets. THC had more variable gene perturbation effects and thus fewer genes were identified across multiple studies. Numbers of studies in which the genes appear as up- or downregulated DEGs are shown in each bar according to the color legend. **B**-**C** Recurrent pathways that were detected based on the enrichment analysis of the DEGs for CBD (**B**) and THC (**C**). The bars are colored based on whether the pathway was up- or downregulated according to the color legend, and the length of the bars indicates the number of exposure datasets in which the pathway was significantly enriched

We next retrieved recurring significant DEGs derived from individual studies, again findings more reproducible DEGs for CBD compared to THC across studies (Fig. 6A, Table S11-12). We note that we did not enforce directional consistency in the DEGs in this analysis due to the well-documented context-specific dynamic and feedback regulation of genes where the same exposure can induce opposite changes between tissues, cell types, dosage, and duration (Ayala-Sumuano et al. 2013; Iravani et al. 2017; Jodynis-Liebert and Kujawska 2020; López-Maury et al. 2008; Noirrit-Esclassan et al. 2021; Piechota et al. 2025; Soumier and Sibille 2014). Among the 30 most frequently detected CBD DEGs, 22 exhibited (> 70%) a consistent direction of regulation, meaning that they were detected as either consistently upregulated or downregulated across datasets (Fig. 6A, Table S11). For example, *SLC30A1*, a zinc transporter (Ryu et al. 2008), was significantly upregulated in 23 CBD datasets and not downregulated in any. *HMOX1* (heme oxygenase 1), a gene involved in oxidative stress response (Casares et al. 2020), was significantly upregulated in 19 datasets while downregulated in only 4 CBD datasets. Some other frequent DEGs play a role in ion metabolism, such as downregulation of *SLC39A10* for zinc transport (Ren et al. 2023) and upregulation of *MT2A* and *MT1E* for metal detoxification and protection against oxidative stress (Schulkens et al. 2014).

In contrast, DEGs associated with THC were less frequently replicated across datasets, and the most recurrent genes appeared only three times across 46 datasets (Fig. 6A, Table S12). *PRXL2A* (peroxiredoxin-like 2 A), an antioxidant protein that protects cells from oxidative stress (Chen et al. 2019), was downregulated in three datasets. Other THC-associated upregulated genes included *BPGM* in red blood cell metabolism, *COL1A2* for collagen synthesis, microtubule-binding protein *FRY*, a potassium channel gene *KCNQ5*, circadian regulator *PER1*, *RALGAPA2* for intracellular signaling, and *XRCC2* for DNA repair.

We also performed pathway enrichment analysis of the significant DEGs for each dataset and summarized the frequency of significant pathways across datasets. Again, THC studies exhibited minimal pathway-level consistency (Fig. 6B, C, Table S13), but CBD datasets showed recurring enriched pathways (Table S14). Consistent CBD pathways include the upregulation of the “metabolic pathway” across 17 studies (Fig. 6B) and the “cellular response to zinc ion” pathway across nine studies (consistent with the reproducibility of DEGs *SLC30A1* and *MT2A*), and the downregulation of cell cycle, mRNA export, cancer, and mismatch repair pathways (Fig. 6C).

#### DEG consistency through rank aggregation

To complement the above analyses, we also performed a meta-analytical Robust Rank Aggregation regardless of regulatory direction (Kolde et al. 2012), revealing 917 THC meta-DEGs and 2505 CBD meta-DEGs with a RRA score < 0.05. For CBD, the top 10 CBD-associated DEGs were *MT2A*, *HMOX1*, *MT1E*, *SLC3A2*, *DDIT3*, *EGR1*, *SLC30A1*, *MT1X*, *MT1F*, and *TRIB3* (Table S15). Nearly all these genes were also among the top 30 recurrent DEGs in individual studies, whereas newly identified meta-DEG, transcriptional regulator EGR1 (O’Donovan et al. 1999), also showed consistent differential expression in 16 individual CBD datasets.

For top 10 meta-DEGs in THC, only *COL1A2* is overlapped with the top DEGs identified by individual occurrences (Fig. 6A, Table S15). Most of the newly identified THC meta-DEGs encode transcriptional regulators, including members of the zinc-finger family (*ZNF544*, *ZNF442*, *ZNF720*, *ZNF300*, *ZNF699*) (Cassandri et al. 2017) and the *FOS* family (*FOS*, *FOSB*) (Milde-Langosch 2005), suggesting that THC exposure predominantly influences transcriptional regulatory pathways. Other top THC meta-DEGs include *VWF* (platelet function and blood clotting) (Ruggeri 2003) and cyclin-dependent kinase inhibitor *CDKN1A* (Ouellet et al. 2006).

### Gene and pathway-level evidence for the endocannabinoid system in CBD and THC transcriptomic signatures

Given the well-established interaction between cannabinoids and the endocannabinoid system (ECS) (Tan et al. 2025; Wu 2019), we anticipated enrichment of ECS-associated genes in the transcriptional signatures of both CBD and THC. To systematically evaluate this, we curated a list of 135 ECS-associated genes using prior literature and established databases, including Gene Ontology Biological Process (GOBP) (Ashburner et al. 2000) and the DisGeNET database (Piñero et al. 2016; 2020) (Table S16). This gene set includes metabolic enzymes involved in endocannabinoid degradation (i.e. *FAAH*, *MGLL*, *ABHD6*, *ABHD12*, *PTGS2*) and biosynthesis (i.e. *NAPEPLD*, *DAGLA*, *DAGLB*) of key ligands, such as 2-arachidonoylglycerol (2-AG) and anandamide (AEA). It also encompasses cannabinoid receptors (i.e. *CRN1*, *CRN2*, *GPR55*, *TRPV1*), neurotransmitter receptors (i.e. *DRD2*, *DRD4*, *HTR1A*, *SSTR4*), or nicotinic acetylcholine receptors (i.e. *CHRNA2*, *CHRNA3*, *CHRNA5*, *CHRNA6*, *CHRNA7*, *CHRNAB3*), endocannabinoid transporters (i.e. *FABP1*, *FABP3*, *FABP5*, *FABP7*), and transcriptional regulators (i.e. *PPARA*, *EP300*, *FOXP2*).

**Fig. 7:**
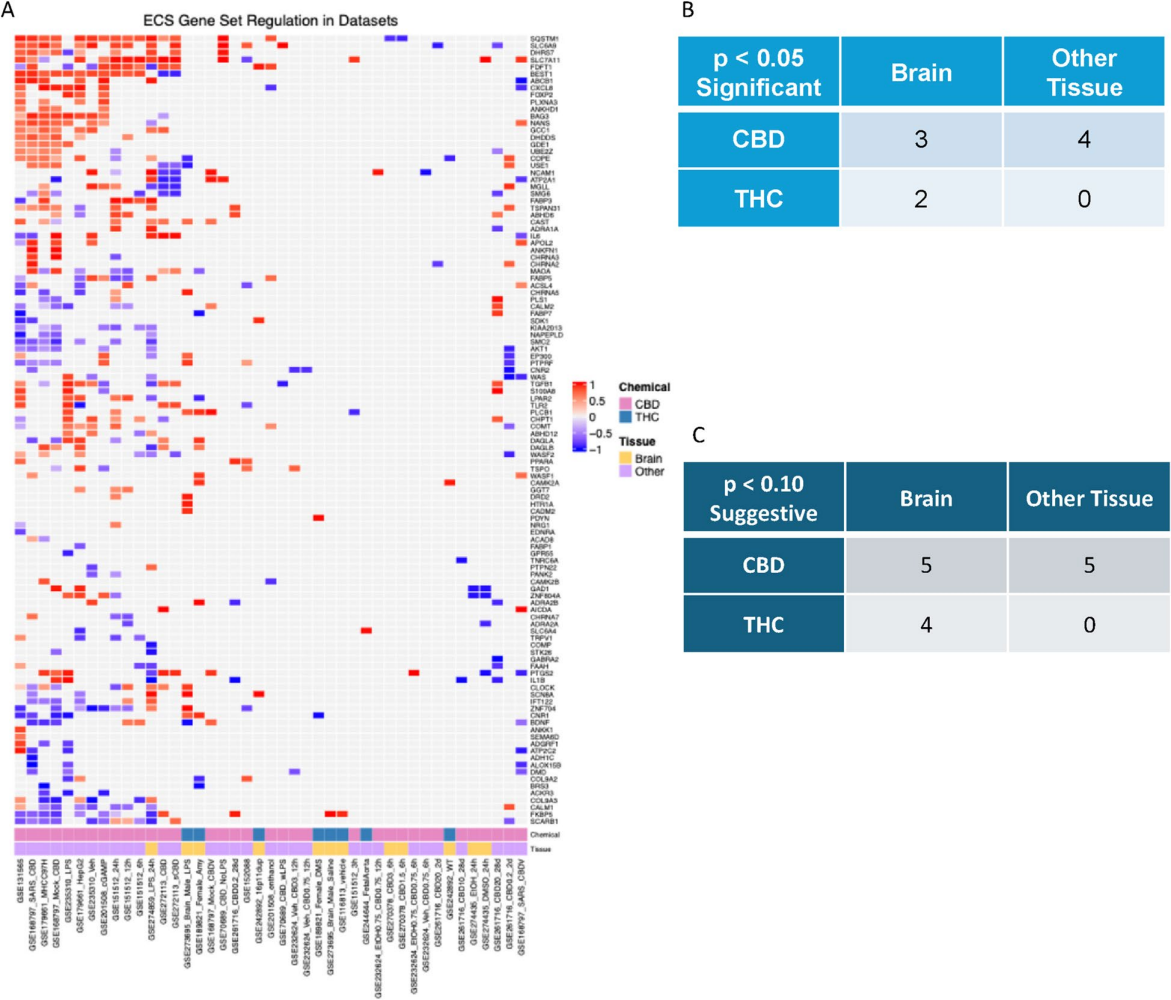
Correlation and overlap analysis reveals the extent of ECS gene involvement in CBD- and THC-induced transcriptome perturbations. **A** The normalized log2 fold changes of the ECS genes detected as significant DEGs in each study are shown in this heatmap, with more highly upregulated genes shown in red hues and more highly downregulated genes shown in blue hues. Clusters of studies with similar directional changes of each gene indicate that the genes are consistently affected by CBD and THC exposure across datasets, and these results indicate ECS gene involvement in CBD- and THC-induced gene perturbations across studies. **B**-**C** Fisher’s exact test was performed to determine if the ECS gene set is significantly enriched in each CBD and THC dataset. The number of datasets derived from brain and peripheral tissue datasets that showed significant enrichment of the ECS gene set at a significant (*p* < 0.05, **B**) and suggestive (*p* < 0.10, **C**) threshold is shown in each table

We examined the regulation of ECS genes identified as DEGs and found that more CBD datasets had significantly regulated ECS genes compared to THC (Fig. 7A). In addition, THC datasets generally exhibited fewer ECS DEGs than CBD datasets. Notably, most THC datasets with significant ECS gene regulation were brain-derived, whereas CBD datasets predominantly originated from peripheral tissues. However, few genes exhibited consistency across studies in significance and direction. This limited degree of coherence suggests context-dependent, tissue-specific regulation of individual genes.

To evaluate whether ECS pathway genes were overrepresented among DEGs, we performed Fisher’s Exact Tests comparing the DEG lists to the ECS gene set. Significance was defined as *p* < 0.05, with *p* < 0.10 considered suggestive (Fig. 7B, C, Table S17). Overall, CBD showed more datasets (across peripheral and central tissues and cell lines) with significant or suggestive enrichment compared to THC (all from brain regions).

In addition, we investigated pathways related to pain and inflammation, which are regulated by the endocannabinoid system (Pertwee 2006). However, no significant enrichment was found for the GOBP “sensory perception of pain pathway” and “response to pain pathway” (Ashburner et al. 2000). However, the inflammatory response pathway was enriched among DEGs from two CBD datasets, both derived from monocyte-derived dendritic cell lines.

### THC- and CBD-induced brain network modeling identifies unique key drivers

**Fig. 8:**
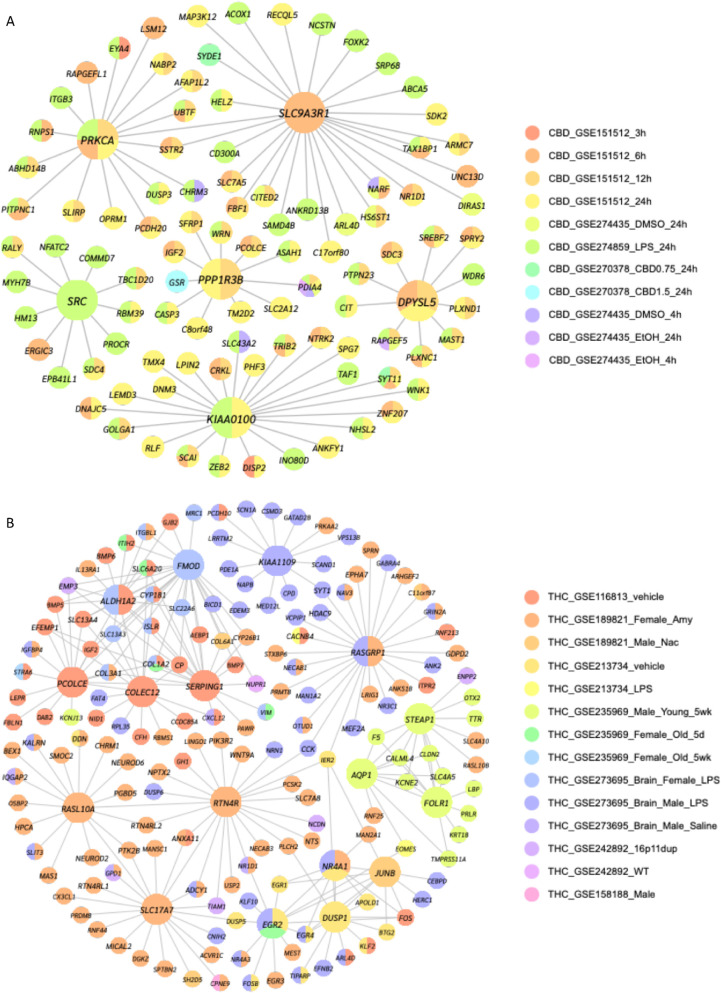
Key driver analysis identifies key regulatory genes in the perturbation effects of CBD and THC. Gene regulatory networks were constructed using key driver analysis from Mergeomics for CBD (**A**) and THC (**B**) brain exposure datasets. The colors of the network nodes indicate datasets in which the gene was a DEG, as described by the color legends. **A** Brain dataset key drivers for CBD were primarily related to neural development and synaptic functions and were consistently detected across datasets from multiple model organisms and brain regions. **B** Brain dataset key drivers for THC were primarily related to neuronal functions, signal transduction, structural integrity, and metabolic functions but were less consistently detected across different datasets, as most were uniquely DEGs in specific studies

We next focused on the brain datasets on THC and CBD to identify key similarities and differences in the regulation of cannabis-induced transcriptomic alterations in the brain. We applied weighted key driver analysis (wKDA) to DEG sets from THC- and CBD-treated brain datasets to identify potential key drivers (KDs) using a Bayesian gene regulatory network previously constructed from dozens of human and mouse brain datasets (Ding et al. 2021; Shu et al. 2016). For CBD-treated brain datasets, 14 significant KDs were identified at FDR < 5% (top 5 shown in Fig. 8A; complete list in Table S18). The KDs in the CBD network were derived from multiple brain cell lines, including mouse brain microglia, rat primary hippocampal neurons, and human neuroblastoma cells (Fig. 8A). Most KDs were involved in neural development and synaptic functions, such as *SH3GL2*, *DPYSL5*, *JPH3*, *SLC17A7*, and *GRIN3B* (*MMT00076709*). Other KDs were associated with signal transduction and kinase activity (*PRKCA*, *TAOK1*, and *ADRBK1*), membrane transport (*SLC9A3R1*), metabolism (*PPP1R3B*), gene regulation (*USF1*), and immune responses and cancer (*SLAMF8*, *SRC*, and *KIAA0100*).

Despite the lack of consistent DEGs and pathways across THC-treated brain datasets, we identified a total of 176 significant KDs based on DEGs from individual datasets (top 5 shown in Fig. 8B; complete list in Table S18), among which *SLC17A7* and *TAOK1* were shared KDs between THC and CBD. Notably, most THC KDs were found to be significant in only a single DEG dataset rather than across multiple datasets (Fig. 8B), suggesting that THC may exert brain region-specific effects on gene regulation. KDs in the THC network were identified from multiple animal models, including the mouse hippocampus, mouse amygdala, mouse microglia, and rat orbitofrontal cortex. A majority of THC-associated KDs were involved in neuronal functions, signal transduction, structural integrity, and metabolic functions. Notably, some KDs with known neuronal roles include *SLC17A7*, *RTN4R*, *RTN4RL1*, *RTN4RL2*, *NEUROD2*, *NEUROD6*, *NGB*, *CAMK2A*, *ADCY1*, *SYT1*, *DLGAP1*, *DLGAP3*, *NTS*, *RGS14*, *GAD2*, and *CNIH3*.

### CBD and THC DEGs are associated with neuropsychiatric disorders while CBD is associated with more metabolic traits

The endocannabinoid system plays a role in various health outcomes, including mood disorders, cardiovascular disease, stroke, cancer, diabetes, autoimmune conditions, and neurological disorders (Lowe et al. 2021). To investigate whether the THC and CBD DEGs identified from our study are enriched for human disease/phenotype variants, we obtained publicly available summary statistics from large-scale Genome-Wide Association Studies (GWAS) for more than 100 diseases or phenotypic traits and performed Marker Set Enrichment Analysis (MSEA) in the Mergeomics package (Ding et al. 2021; Shu et al. 2016).

**Fig. 9:**
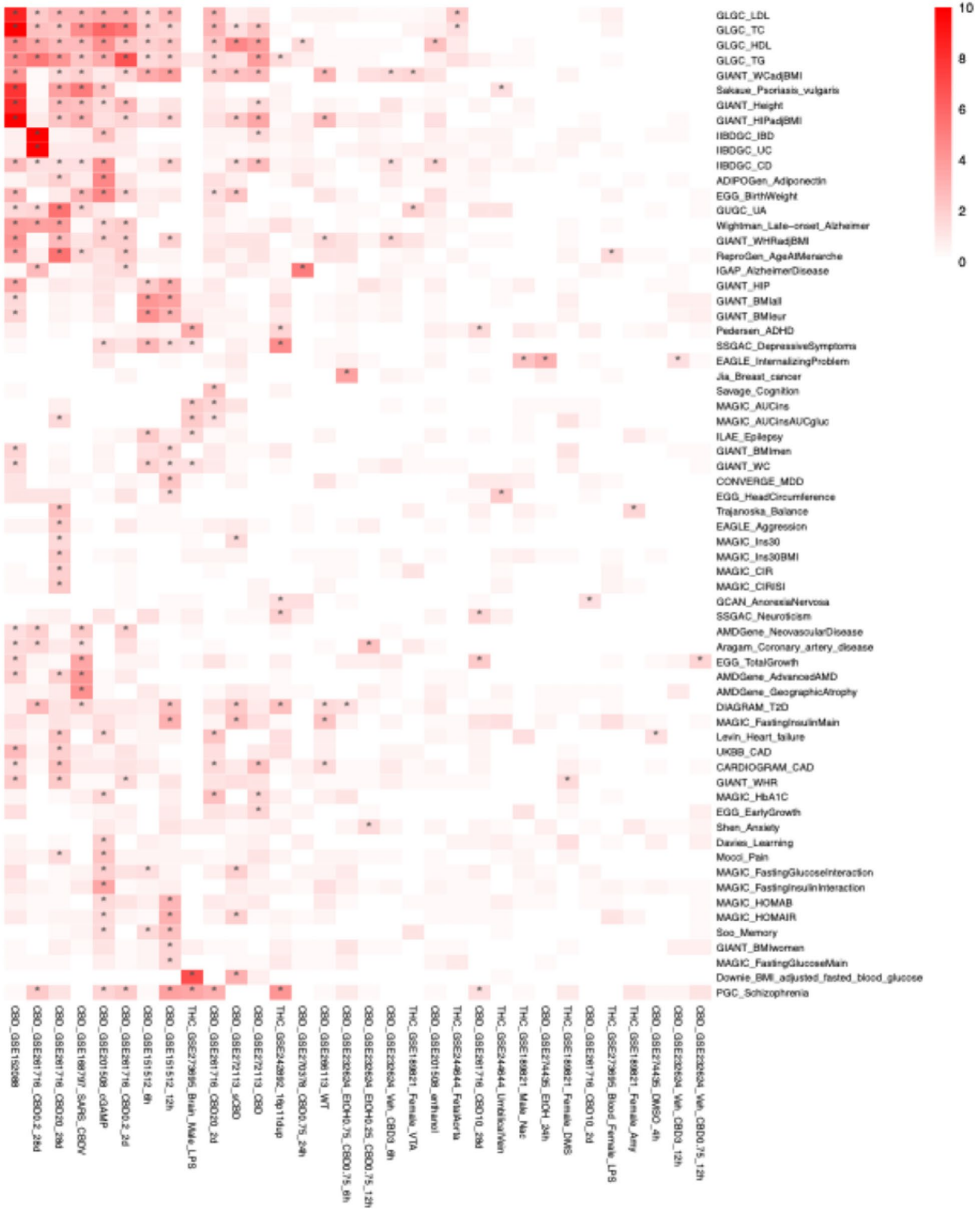
Marker set enrichment analysis (MSEA) reveals associations between cannabinoid exposure and psychiatric, metabolic diseases. MSEA from Mergeomics was performed on the DEGs from each dataset to identify disease associations based on marker genes for each disease. Only diseases or traits with at least one significantly associated dataset are shown. More highly associated diseases are shown with redder hues, and significant associations are starred. Psychiatric conditions, including schizophrenia and depression, and metabolic conditions, such as type 2 diabetes, were detected as significant associations with both CBD and THC exposure

DEGs from both cannabinoids showed significant associations with Schizophrenia, type 2 diabetes (T2D), and depressive symptoms (Fig. 9). In addition, CBD transcriptomic signatures exhibited broader associations with lipid metabolism, including low-density lipoprotein (LDL), high-density lipoprotein (HDL), total cholesterol (TC), and triglycerides (TG), as well as with body composition traits such as waist circumference (WCadjBMI), hip circumference (HIPadjBMI), waist-to-hip ratio (WHRadjBMI), and height. In contrast, THC was linked only to WCadjBMI. Beyond metabolic and anthropometric traits, CBD was associated with Crohn’s disease (CD) and coronary artery disease (CAD).

To identify the genes most strongly linked to disease-associated SNPs, we examined the top genes from the significantly enriched datasets (Fig. S6A, S6B, S6C, S6D). In the CBD datasets, key associations for CD included tumor suppressor *CYLD* (Massoumi 2011) and cytosolic receptor *NOD2* (Fig. S6A). Mutations or altered expression of *NOD2* have been observed in CD patients (Negroni et al. 2018), and genetic studies have identified *NOD2* locus polymorphisms and an independent involvement of the neighboring gene *CYLD* in CD (Elding et al. 2011). For T2D, a key association for CBD DEGs was the Wnt signaling pathway transcription factor *TCF7L2* (Fig. S6C). Carrying two copies of a common variant in this gene is associated with an approximately twofold increase in T2D risk (Bosque-Plata et al. 2021; Weedon 2007). CBD DEGs CDKN2A and CDKN2B were also among the top CAD-associated GWAS candidates, which are located adjacent to the lead CAD-linked SNP on the 9p21 locus (Zivotić et al. 2019) (Fig. S6D). In contrast, THC showed more moderate levels of association between DEGs and disease-related SNPs. Of note, this analysis mainly focuses on overlaps between CBD/THC DEGs with genetic associations of diseases and does not implicate increases or decreases in disease risks.

## Discussion

In this study, we systematically analyzed over 100 publicly available transcriptomic datasets to understand the molecular effects of THC and CBD across species, tissue types, and experimental platforms. We found that datasets for these two cannabinoids showed less coherent patterns compared to other exposures such as PFOA, BPA, and estradiol, suggesting context-specific transcriptomic responses. We also identified DEGs, pathways, network regulators, and diseases/phenotypes associated with the significant DEGs. Our findings highlight differential transcriptional responses between THC and CBD, with CBD demonstrating better consistency across studies, species, and central and peripheral tissue types than THC, which shows more dataset-specific effects in brain regions.

Our broad dimensionality reduction analysis revealed that CBD and THC datasets were more scattered in contrast with other well-studied chemicals PFOA, BPA, and estradiol, which displayed stronger internal similarity across datasets. In addition, neither THC nor CBD datasets formed larger clusters in the correlation analyses across species and tissues. Indeed, study design factors such as species, tissue, sex and experimental platforms all showed strong influence on the DEGs detected. These results highlight the challenge of defining a universal transcriptional signature for cannabinoids and align with previous literature indicating that cannabinoid effects depend on tissue, developmental stage, sex, and exposure conditions (Zuo et al. 2022; Shapira et al. 2023; Bilkei-Gorzo et al. 2017; Martin 1986).

Comparing CBD to THC, our analyses on the DEGs and enriched pathways across datasets indicate that CBD has a more consistent gene regulatory signature than THC across datasets, and the genes and pathways also agree with CBD’s well-characterized antioxidant, anti-inflammatory, and neuroprotective effects (Al-Khazaleh et al. 2024; Atalay et al. 2019). In particular, two major CBD-associated gene clusters (CBD Cluster 1 and CBD Cluster 4) exhibited hundreds to thousands of consistent DEGs, with both CBD clusters exhibiting upregulation in negative regulation of growth and cellular response to zinc ions. CBD was associated with higher frequency of consistent DEGs across datasets, such as *HMOX1*, *SLC30A1*, *MT2A*, and *SLC39A10*. Previous in vitro studies have identified *HMOX1* as the most upregulated gene and protein following CBD treatment via CBD-induced nuclear export and proteasomal degradation of transcriptional repressor BACH (Casares et al. 2020). Another study suggests that CBD may influence *HMOX1* levels through the Nrf2 pathway, which regulates antioxidant defenses (Ekiner et al. 2022). These indicate that *HMOX1* is linked to the antioxidant and anti-inflammatory properties of CBD (Baswan et al. 2020). Notably, pathways related to metabolic regulation and cellular response to zinc ion were recurrently upregulated. Supporting this finding, a previous study in BV-2 microglial cells reported that CBD upregulated zinc-related genes (*Mt2*, *Ndrg1*, *Mmp23*) and zinc transporters *SLC30A1* and *SLC39A4* while downregulating *SLC39A10* and *ZFP472* (Juknat et al. 2012). Another study in autoimmune T cells found that CBD suppressed pro-inflammatory genes and enhanced oxidative stress-related genes, including *SLC30A1* (Kozela et al. 2016). We now expand this finding to a much broader range of tissues and experimental conditions. Together, these results suggest that modulation of zinc homeostasis may be a key mechanism through which CBD exerts its antioxidant and anti-inflammatory effects. The cell cycle and cancer pathway were top downregulated pathways across CBD-associated datasets. Prior studies have shown that CBD can arrest the cell cycle in the G0/G1 phase and promote cell death in gastric cancer cell lines, such as SGC-7901 (Pagano et al. 2021). Our study of around 50 datasets highlighting this pathway across studies further highlight the potential anti-cancer role of CBD in addition to its metabolic and immunomodulatory effects.

In contrast, THC datasets exhibited lower levels of gene and pathway consistency across datasets, reinforcing its context-dependent effects. The weaker transcriptional consistency of THC may stem from its complex pharmacodynamics, particularly its biphasic effects on neural and immune signaling. Despite the lack of strong gene level consistency, the recurrent DEGs are involved in central and peripheral nervous system functions. For example, among the most consistent DEGs induced by THC was *EGR2*, which is associated with neuropathy and congenital hypomyelination of peripheral neurons (Nagarajan et al. 2001). A number of THC target DEGs were also associated with synaptic signaling in the brain, and the onset of epilepsy and intellectual deficits, including *RPH3A* (Pavinato et al. 2023), *KCNQ5* (Wei et al. 2022), and *FRY* (Paulraj et al. 2019). Further, *PER1* was among the most consistently detected DEGs, which is a primary circadian pacemaker which has implications on human behavior and cognition (Lim et al. 2012). *FRY* and *EGR2* are associated with neuronal irregularities in region-specific targets (e.g. the cerebellum for *FRY* and peripheral neurons for *EGR2*). One possible explanation for the highly variable effects of THC lies in its high lipid solubility (Chayasirisobhon 2020), which may influence its distribution across datasets depending on adipose content and tissue composition. Additionally, physiological barriers such as the blood–brain and blood-testicular barrier restrict THC accumulation in the brain and testes during acute exposure, and similar protective mechanisms may exist in other tissues as well (Huestis 2007). These factors together may contribute to the heterogeneous effects of THC across individuals and biological contexts.

We also observed differential network properties of CBD and THC in the brain. The CBD network had only 14 KDs orchestrating DEGs across datasets. By contrast, the THC network contained over 100 KDs, most of which were significant for only one DEG dataset, further supporting that THC induces region-specific effects in the brain. Additionally, only two KDs, *SLC17A7* and *TAOK1*, were shared between them. *SLC17A7* encodes vesicular glutamate transporter 1 (*VGLUT1*), which facilitates glutamatergic neurotransmission (Reimer 2013). Cannabinoids have been shown to influence glutamatergic signaling: CBD reduces neuronal activation in VGLUT + neurons (Nedelescu et al. 2022), while cannabinol upregulates genes associated with glutamatergic synaptic function (Trainito et al. 2024). *TAOK1* encodes a serine/threonin-protein that functions as a MAP kinase kinase kinase (MAP3K) and regulates MAPK signaling cascade (Fang et al. 2020). Although direct evidence linking cannabis treatment to *TAOK1* regulation is lacking, THC has been shown to modulate microRNAs that target mRNAs of proteins involved in MAPK signaling, including *TAOK1* (Simon et al. 2016). The limited overlap in KDs between the two networks highlights the distinct molecular and cellular mechanisms through which THC and CBD exert their effects in the brain and is in line with our broader findings that THC and CBD regulate largely non-overlapping genes and pathways across tissues.

While the endocannabinoid system (ECS) is widely recognized as a primary target of phytocannabinoids such as THC and CBD (Tan et al. 2025), our multi-dataset analysis revealed that ECS-associated genes were regulated inconsistently across studies, both in direction and magnitude. These inconsistencies might reflect tissue-specific expression of ECS components as well as distinct pharmacological profiles of THC and CBD. For example, in terms of binding affinity, THC acts as a partial agonist at both cannabinoid receptors, while CBD exhibits negligible affinity for cannabinoid receptor 1 (CB1) and functions as a partial agonist at cannabinoid receptor 2 (CB2) (Zou and Kumar 2018; Shahbazi et al. 2020). Despite this gene-level variability, pathway-level analysis demonstrated that the ECS-related gene set was significantly enriched in brain-derived datasets for THC and in both central and peripheral datasets (immune-related cell types and liver tissue) for CBD (Fig. 6B, C). This divergence likely reflects differences in receptor affinity and expression patterns. CB1 is abundantly expressed in the brain and to a lesser extent in select peripheral tissues, whereas CB2 is predominantly found in immune cells and exhibits limited expression in the central nervous system (Schönke et al. 2020). Thus, THC’s higher binding affinity to CB1 compared to CB2 explains its brain-focused transcriptomic effects (Schönke et al. 2020), while CBD’s lack of CB1 binding and moderate activity at CB2 likely underlies its broader effects across peripheral and immune contexts.

To further investigate the potential health implications of cannabis exposure, we assessed whether gene expression profiles associated with THC and CBD were enriched for genetic markers of human diseases. CBD-responsive genes showed significant enrichment for markers related to cholesterol and lipid metabolism traits, aligning with prior studies indicating that CBD can modulate lipid profiles and holds therapeutic potential for managing lipid disorders (Wiciński et al. 2023). CBD was also significantly associated with anthropometric traits, such as waist circumference, hip circumference, and height. A systematic review across multiple databases and registries reported that while cannabis use is generally associated with reductions in weight, waist circumference, and BMI, CBD specifically has been linked to increased body fat (Reis et al. 2024).

Both CBD and THC show association with Schizophrenia, type 2 diabetes (T2D), and depressive symptoms. The relationship between cannabis use and depression is particularly complex and potentially bidirectional. Studies relying on self-reported depressive symptoms suggest mixed short-term effects of cannabis use: a minority (20%) with increased depression and a majority (64%) with decreased depression (Li et al. 2020). However, a meta-analysis of 15 studies found that even a single THC administration induces depression and anxiety with large effect sizes (Hindley et al. 2020). Conversely, extended cannabis abstinence has been associated with significant improvements of depressive symptoms (Lucatch et al. 2020). Longitudinal data also suggest a bidirectional or reinforcing relationship, as baseline depression has been significantly associated with increased THC use in e-cigarettes 12 months later (Clendennen et al. 2023).

CBD and other non-psychoactive cannabinoids have been investigated in human clinical trials for their potential therapeutic benefits in T2D and Schizophrenia. Indeed, a randomized clinical trial showed that CBD reduced circulating resistin, a hormone associated with insulin resistance, and increased glucose-dependent insulinotropic peptide (GIP), which plays a role in preserving pancreatic β-cell function in T2D patients (Jadoon et al. 2016). CBD has also demonstrated beneficial effects and a safety profile in Schizophrenia, where patients treated with CBD for six weeks demonstrated lower levels of positive psychotic symptoms (McGuire et al. 2018). In another randomized clinical trial comparing CBD with the antipsychotic amisulpride, both treatments led to significant clinical improvement; however, CBD had a markedly superior side-effect profile (Leweke et al. 2012). Thus, our disease relevance results, supported by human clinical evidence, highlight the broad therapeutic potential of cannabinoids, especially CBD, in metabolic and psychiatric diseases and underscore the relevance of cannabinoid-responsive genes in the genetic architecture of complex traits.

Here, we have conducted a comprehensive, multispecies, multitissue investigation of the metabolic effects of THC and CBD. One of the primary strengths of our study was the use of highly heterogeneous datasets, with variations in experimental design, dosing regimens, and sample characteristics. This allowed us to consider cannabinoid exposure in a number of biological contexts, including in co-occurrence with diseases, on top of exposure to other relevant chemical agents, and in different tissues or regions within those tissues (e.g. several different brain regions were covered by our datasets). Meta-analysis across species allows us to consider the maximal amount of data from translatable model organisms so we can draw human-relevant conclusions. Despite the comprehensive nature of our analysis, however, some important limitations should be acknowledged. First, while we applied normalization techniques to minimize batch effects, residual variability may have influenced our findings. However, the same procedures were applied to the datasets for the reference chemicals, where coherent clustering and coherence across datasets were found. Therefore, our findings are less likely due to technical artifacts but are more likely the results of intrinsic activities of the compounds examined. Second, the sample sizes for cannabinoid studies tend to be small, with most groups consisting of only around three replicates and many datasets did not reveal significant DEGs. This limitation reflects the current state of the field and underscores the need for larger, more coordinated genomic studies. We adopted a meta-analytic strategy to integrate signatures across studies, enhancing robustness through aggregated evidence. Third, the use of bulk transcriptomic data precludes cell type-specific resolution, which is critical for understanding the differential effects of cannabinoids on distinct cell populations. Future studies employing single-cell RNA sequencing or spatial transcriptomics, when datasets are available, could provide deeper insights into the cellular specificity of cannabinoid-induced gene expression changes. Additionally, while we focused on gene expression data, post-transcriptional and epigenetic modifications likely play a crucial role in cannabinoid-mediated effects. Integrating multi-omics approaches, including proteomics and metabolomics, could enhance our understanding of cannabinoid biology. Last but not least, the existing datasets for THC and CBD are not balanced, with more in vitro data for CBD and more in vivo data for THC, which may confound some of our conclusions. Although sensitivity analysis to compare the two compounds in subsets of datasets with more comparable conditions confirmed the main conclusions, there is a need for more balanced and comprehensive studies to increase dataset coverage and density for cannabinoids to validate our findings. Additional gene perturbation studies are also needed to validate the network regulators identified.

In summary, this study provides a comprehensive transcriptomic analysis of THC and CBD across species and tissue types. Our findings demonstrate that CBD exhibits greater transcriptional consistency across datasets compared to THC, and affects metabolic, cell cycle, and inflammatory pathways. In contrast, THC-induced gene expression changes are more context-dependent. These insights have important implications for the therapeutic use of cannabinoids and suggests the need for more careful monitoring of the biological effects of THC. As the legalization and medicinal use of cannabis continues to expand, a deeper understanding of its molecular mechanisms will be critical for optimizing its clinical applications while minimizing potential risks.

## Supplementary Information

Supplementary Material 1: Supplement Table 1. Summary of publicly available cannabis-related transcriptomic datasets. Supplement Table 2. Summary of cannabis-related and reference transcriptomic datasets. Supplement Table 3. Summary of number of DEGs in each dataset. Supplement Table 4. Multi-way ANOVA of chemical and experimental factors on DEG counts. Supplement Table 5. THC Cluster 1 cluster-level DEG summary. Supplement Table 6. THC Cluster 2 cluster-level DEG summary. Supplement Table 7. CBD Cluster 1 cluster-level DEG summary. Supplement Table 8. CBD Cluster 4 cluster-level DEG summary. Supplement Table 9. CBD Cluster 2 cluster-level DEG summary. Supplement Table 10. CBD Cluster 3 cluster-level DEG summary. Supplement Table 11. Frequency of DEGs across CBD datasets. Supplement Table 12. Frequency of DEGs across THC datasets. Supplement Table 13. Frequency of pathways across THC datasets. Supplement Table 14. Frequency of pathways across CBD datasets. Supplement Table 15. *p*-value aggregated meta-DEGs across THC and CBD datasets. Supplement Table 16. ECS-associated genes and reference sources. Supplement Table 17. Fisher’s exact tests results between DEG lists and ECS-associated genes. Supplement Table 18. Key driver analysis results for THC and CBD datasets.

Supplementary Material 2: Supplementary Figures.

## Data Availability

No datasets were generated or analysed during the current study.
