# Supplementary material for: Comparative analysis of 105 datasets across species and tissues reveals differential transcriptomic responses to cannabinoids THC and CBD
Source: J Cannabis Res. 2025 Dec 16;8:13. doi: 10.1186/s42238-025-00361-0 (PMC12821297; doi:10.1186/s42238-025-00361-0)
Supplement: Supplementary file 2 — Supplementary Material 2: Supplementary Figures. [file 42238_2025_361_MOESM2_ESM.pptx]

## Slide 1
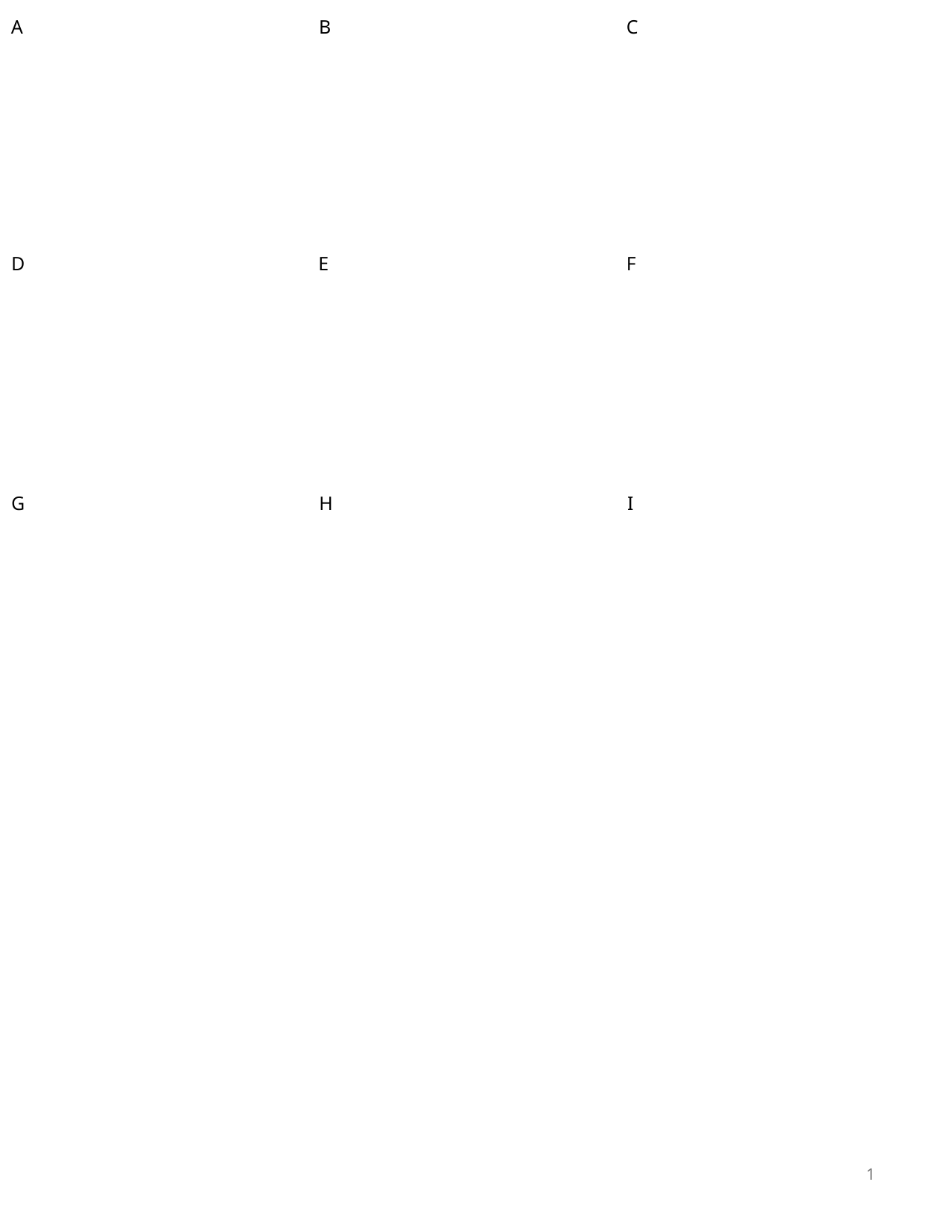

B
C
A
F
E
D
I
G
H
1

## Slide 2
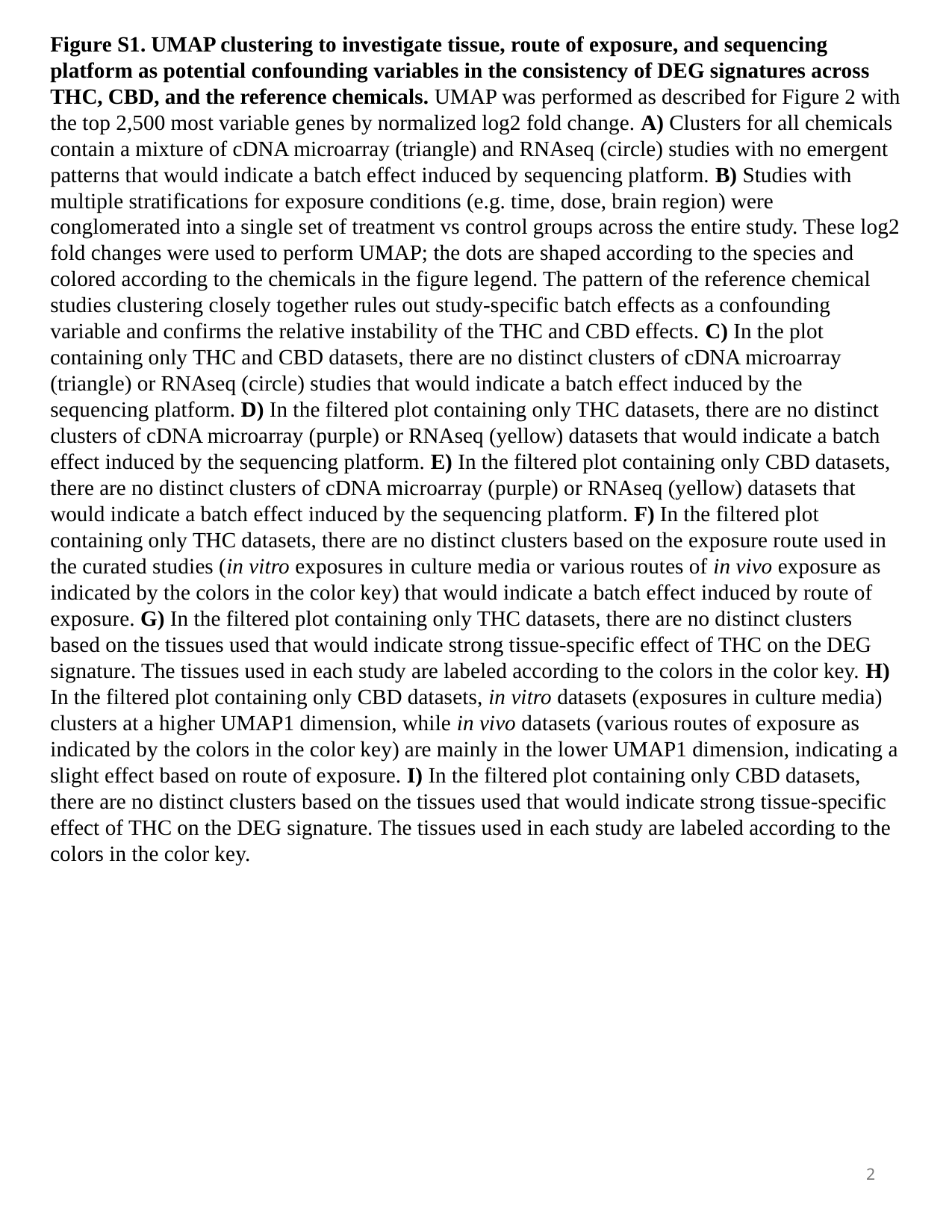

Figure S1. UMAP clustering to investigate tissue, route of exposure, and sequencing platform as potential confounding variables in the consistency of DEG signatures across THC, CBD, and the reference chemicals. UMAP was performed as described for Figure 2 with the top 2,500 most variable genes by normalized log2 fold change. A) Clusters for all chemicals contain a mixture of cDNA microarray (triangle) and RNAseq (circle) studies with no emergent patterns that would indicate a batch effect induced by sequencing platform. B) Studies with multiple stratifications for exposure conditions (e.g. time, dose, brain region) were conglomerated into a single set of treatment vs control groups across the entire study. These log2 fold changes were used to perform UMAP; the dots are shaped according to the species and colored according to the chemicals in the figure legend. The pattern of the reference chemical studies clustering closely together rules out study-specific batch effects as a confounding variable and confirms the relative instability of the THC and CBD effects. C) In the plot containing only THC and CBD datasets, there are no distinct clusters of cDNA microarray (triangle) or RNAseq (circle) studies that would indicate a batch effect induced by the sequencing platform. D) In the filtered plot containing only THC datasets, there are no distinct clusters of cDNA microarray (purple) or RNAseq (yellow) datasets that would indicate a batch effect induced by the sequencing platform. E) In the filtered plot containing only CBD datasets, there are no distinct clusters of cDNA microarray (purple) or RNAseq (yellow) datasets that would indicate a batch effect induced by the sequencing platform. F) In the filtered plot containing only THC datasets, there are no distinct clusters based on the exposure route used in the curated studies (in vitro exposures in culture media or various routes of in vivo exposure as indicated by the colors in the color key) that would indicate a batch effect induced by route of exposure. G) In the filtered plot containing only THC datasets, there are no distinct clusters based on the tissues used that would indicate strong tissue-specific effect of THC on the DEG signature. The tissues used in each study are labeled according to the colors in the color key. H) In the filtered plot containing only CBD datasets, in vitro datasets (exposures in culture media) clusters at a higher UMAP1 dimension, while in vivo datasets (various routes of exposure as indicated by the colors in the color key) are mainly in the lower UMAP1 dimension, indicating a slight effect based on route of exposure. I) In the filtered plot containing only CBD datasets, there are no distinct clusters based on the tissues used that would indicate strong tissue-specific effect of THC on the DEG signature. The tissues used in each study are labeled according to the colors in the color key.
2

## Slide 3
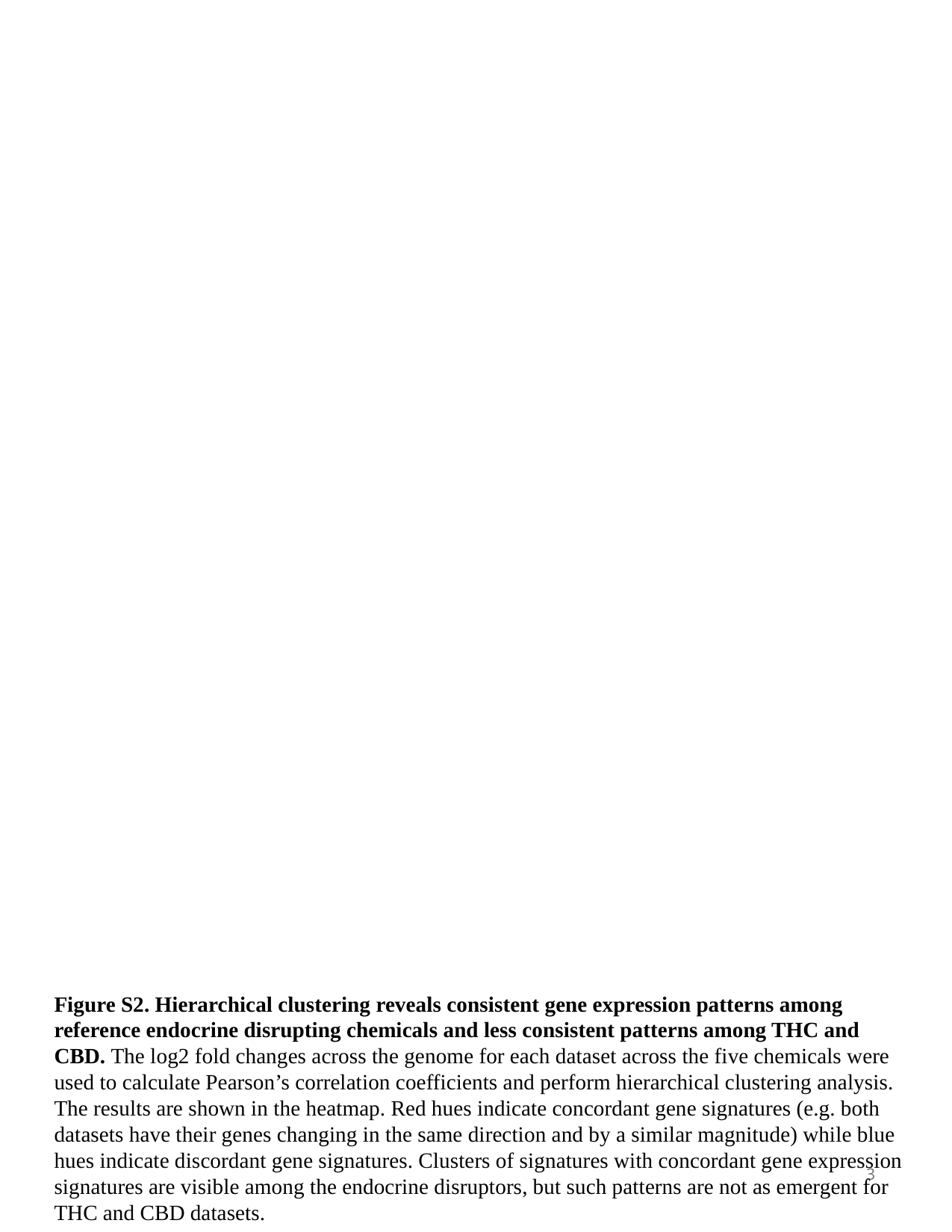

Figure S2. Hierarchical clustering reveals consistent gene expression patterns among reference endocrine disrupting chemicals and less consistent patterns among THC and CBD. The log2 fold changes across the genome for each dataset across the five chemicals were used to calculate Pearson’s correlation coefficients and perform hierarchical clustering analysis. The results are shown in the heatmap. Red hues indicate concordant gene signatures (e.g. both datasets have their genes changing in the same direction and by a similar magnitude) while blue hues indicate discordant gene signatures. Clusters of signatures with concordant gene expression signatures are visible among the endocrine disruptors, but such patterns are not as emergent for THC and CBD datasets.
3

## Slide 4
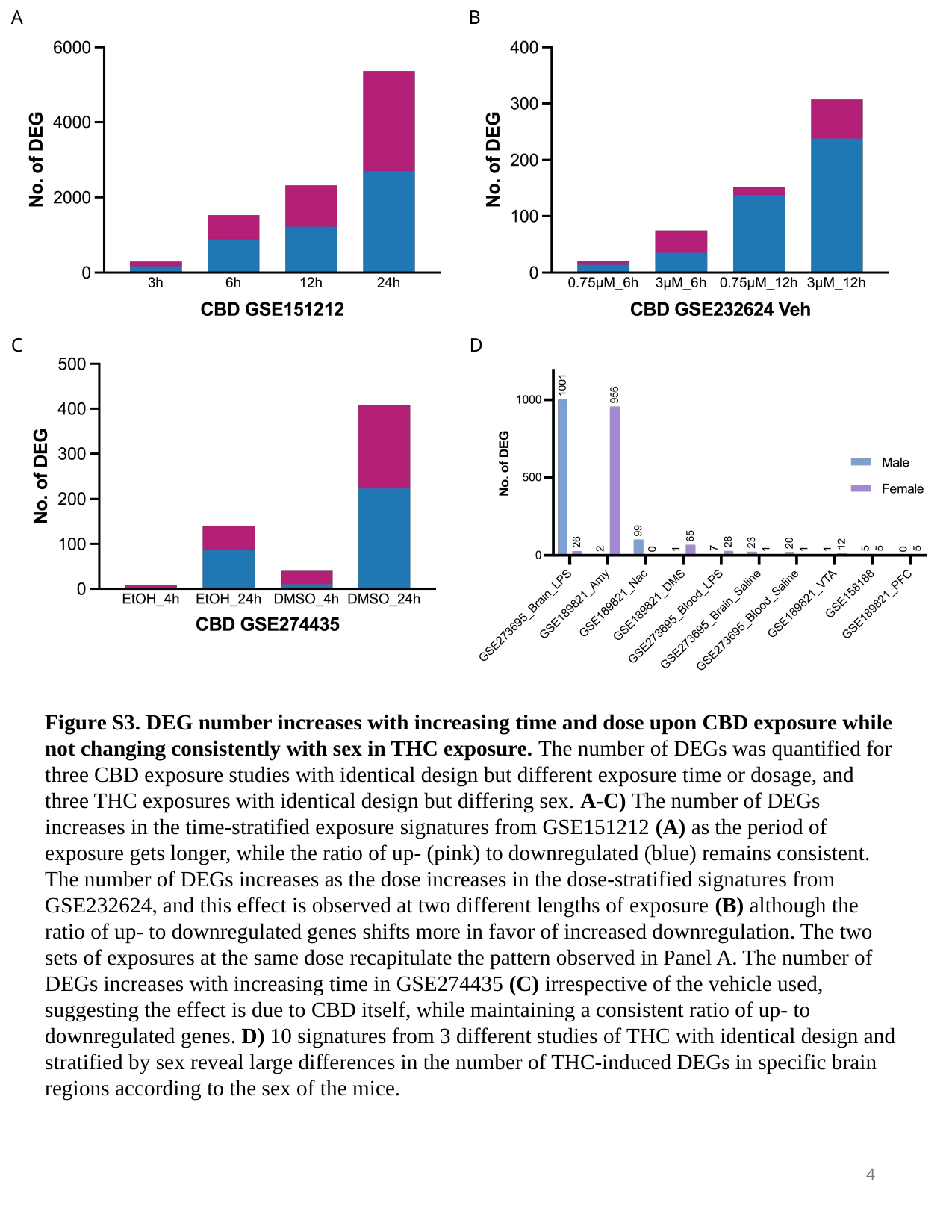

A
B
C
D
Figure S3. DEG number increases with increasing time and dose upon CBD exposure while not changing consistently with sex in THC exposure. The number of DEGs was quantified for three CBD exposure studies with identical design but different exposure time or dosage, and three THC exposures with identical design but differing sex. A-C) The number of DEGs increases in the time-stratified exposure signatures from GSE151212 (A) as the period of exposure gets longer, while the ratio of up- (pink) to downregulated (blue) remains consistent. The number of DEGs increases as the dose increases in the dose-stratified signatures from GSE232624, and this effect is observed at two different lengths of exposure (B) although the ratio of up- to downregulated genes shifts more in favor of increased downregulation. The two sets of exposures at the same dose recapitulate the pattern observed in Panel A. The number of DEGs increases with increasing time in GSE274435 (C) irrespective of the vehicle used, suggesting the effect is due to CBD itself, while maintaining a consistent ratio of up- to downregulated genes. D) 10 signatures from 3 different studies of THC with identical design and stratified by sex reveal large differences in the number of THC-induced DEGs in specific brain regions according to the sex of the mice.
4

## Slide 5
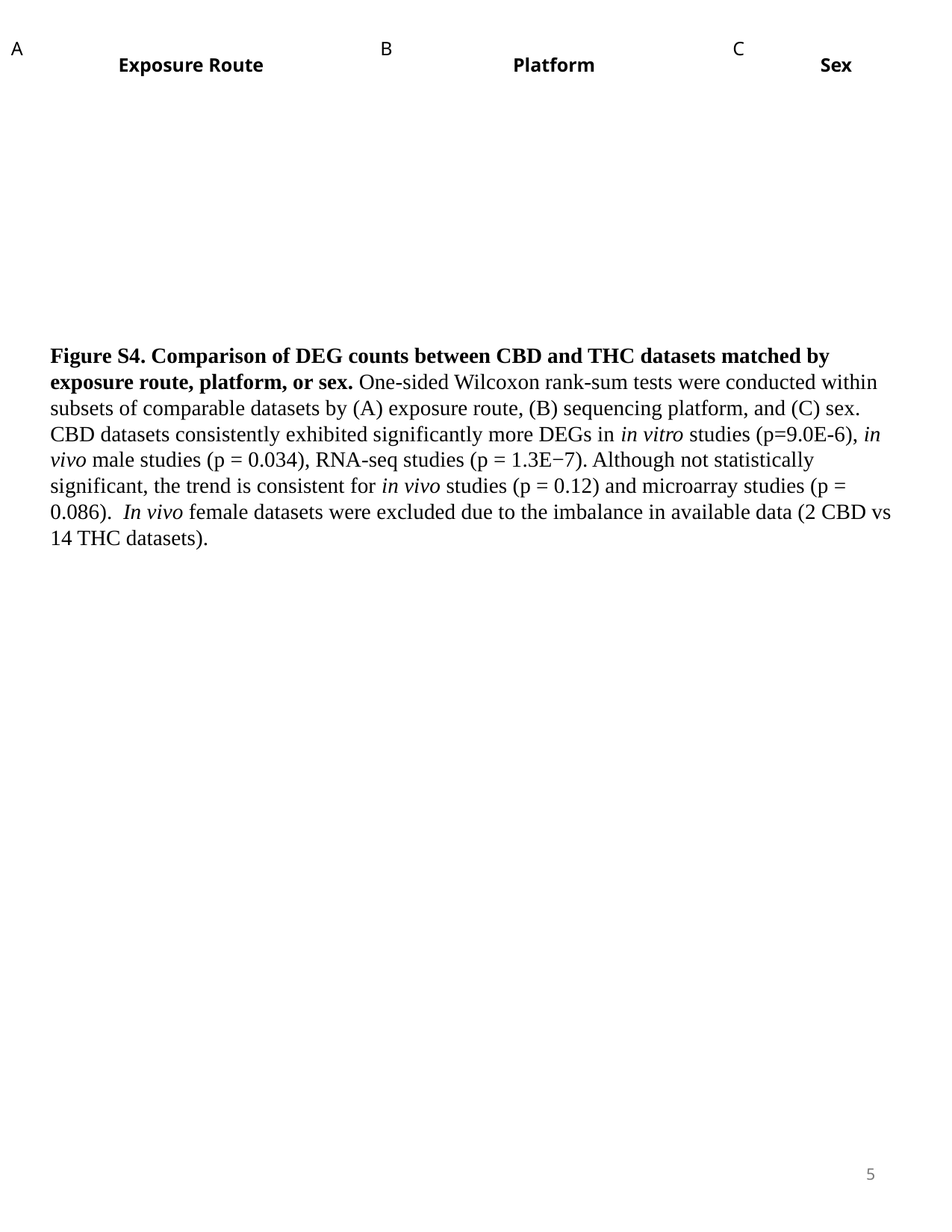

A
B
C
Exposure Route
Platform
Sex
Figure S4. Comparison of DEG counts between CBD and THC datasets matched by exposure route, platform, or sex. One-sided Wilcoxon rank-sum tests were conducted within subsets of comparable datasets by (A) exposure route, (B) sequencing platform, and (C) sex. CBD datasets consistently exhibited significantly more DEGs in in vitro studies (p=9.0E-6), in vivo male studies (p = 0.034), RNA-seq studies (p = 1.3E−7). Although not statistically significant, the trend is consistent for in vivo studies (p = 0.12) and microarray studies (p = 0.086).  In vivo female datasets were excluded due to the imbalance in available data (2 CBD vs 14 THC datasets).
5

## Slide 6
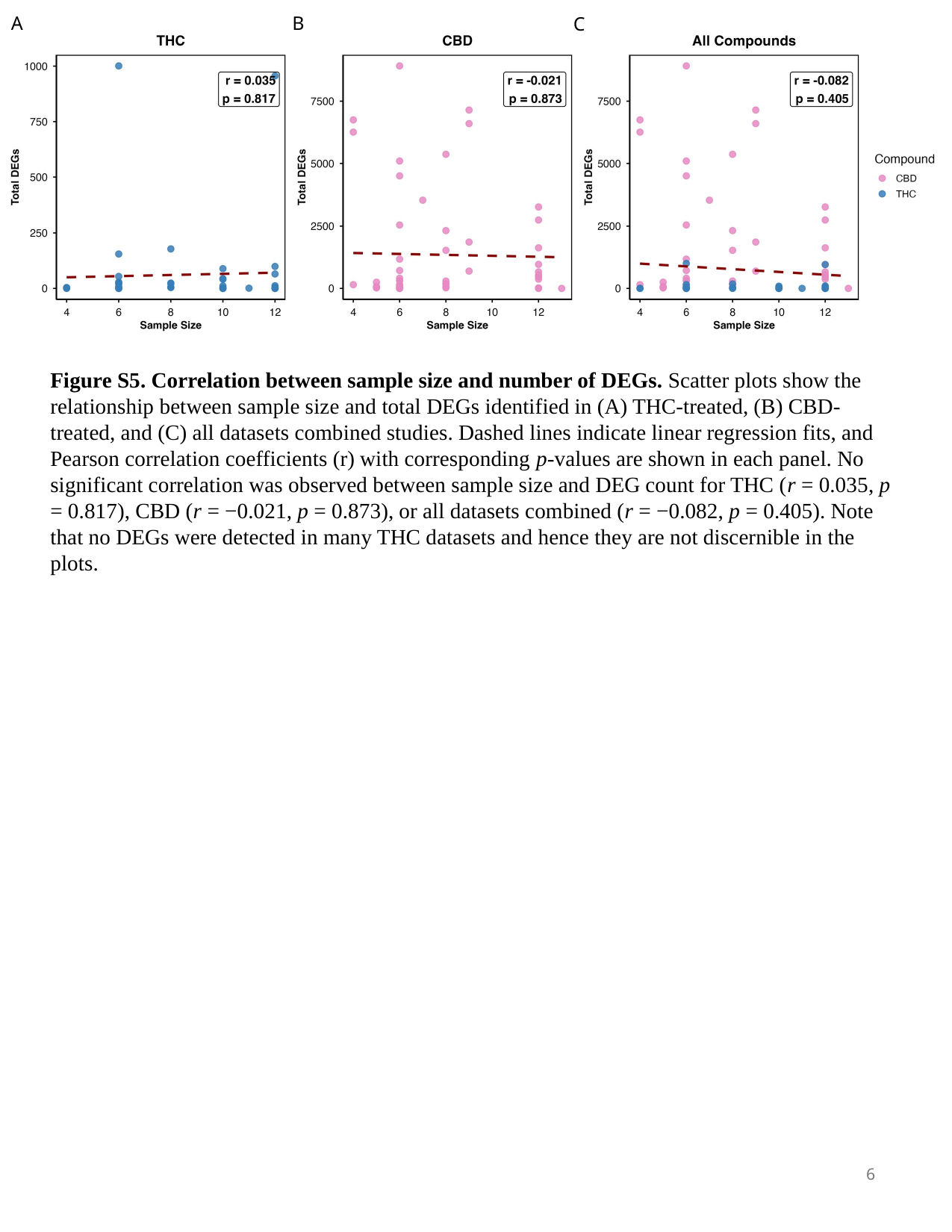

A
B
C
Figure S5. Correlation between sample size and number of DEGs. Scatter plots show the relationship between sample size and total DEGs identified in (A) THC-treated, (B) CBD-treated, and (C) all datasets combined studies. Dashed lines indicate linear regression fits, and Pearson correlation coefficients (r) with corresponding p-values are shown in each panel. No significant correlation was observed between sample size and DEG count for THC (r = 0.035, p = 0.817), CBD (r = −0.021, p = 0.873), or all datasets combined (r = −0.082, p = 0.405). Note that no DEGs were detected in many THC datasets and hence they are not discernible in the plots.
6

## Slide 7
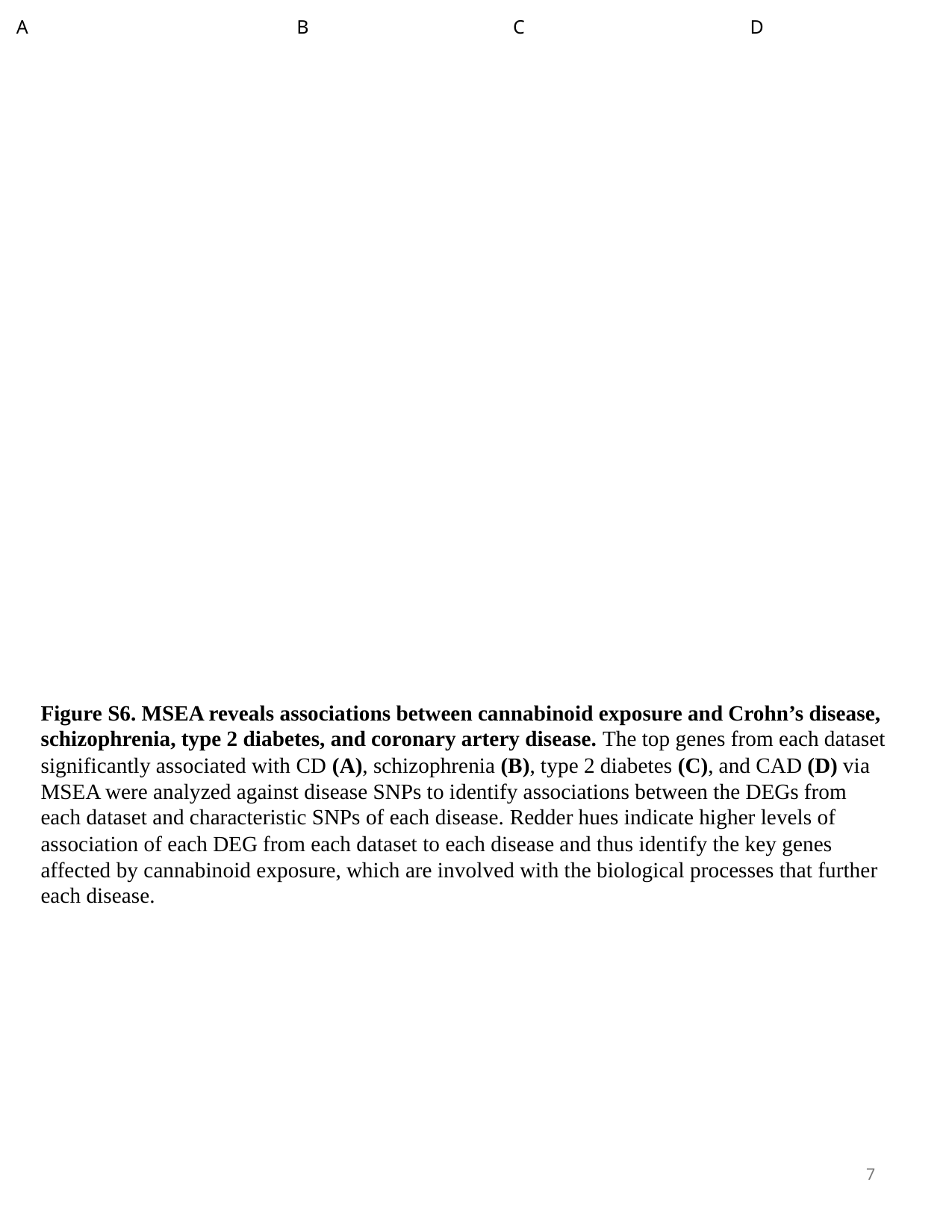

A
B
C
D
Figure S6. MSEA reveals associations between cannabinoid exposure and Crohn’s disease, schizophrenia, type 2 diabetes, and coronary artery disease. The top genes from each dataset significantly associated with CD (A), schizophrenia (B), type 2 diabetes (C), and CAD (D) via MSEA were analyzed against disease SNPs to identify associations between the DEGs from each dataset and characteristic SNPs of each disease. Redder hues indicate higher levels of association of each DEG from each dataset to each disease and thus identify the key genes affected by cannabinoid exposure, which are involved with the biological processes that further each disease.
7
